# Connecting the molecular function of microRNAs to cell differentiation dynamics

**DOI:** 10.1098/rsif.2019.0437

**Published:** 2019-09-25

**Authors:** Russell Posner, Reinhard Laubenbacher

**Affiliations:** 1 Center for Quantitative Medicine, UConn Health, Farmington, CT, USA; 2 The Jackson Laboratory for Genomic Medicine, Farmington, CT, USA

**Keywords:** gene regulatory networks, microRNAs, control of differentiation

## Abstract

MicroRNAs form a class of short, non-coding RNA molecules which are essential for proper development in tissue-based plants and animals. To help explain their role in gene regulation, a number of mathematical and computational studies have demonstrated the potential canalizing effects of microRNAs. However, such studies have typically focused on the effects of microRNAs on only one or a few target genes. Consequently, it remains unclear how these small-scale effects add up to the experimentally observed developmental outcomes resulting from microRNA perturbation at the whole-genome level. To answer this question, we built a general computational model of cell differentiation to study the effect of microRNAs in genome-scale gene regulatory networks. Our experiments show that in large gene regulatory networks, microRNAs can control differentiation time without significantly changing steady-state gene expression profiles. This temporal regulatory role cannot be naturally replicated using protein-based transcription factors alone. While several microRNAs have been shown to regulate differentiation time *in vivo*, our findings provide a new explanation of how the cumulative molecular actions of individual microRNAs influence genome-scale cellular dynamics. Taken together, these results may help explain why tissue-based organisms exclusively depend on miRNA-mediated regulation, while their more primitive counterparts do not.

## 1. Introduction

MicroRNAs (miRNAs) are a class of short, 16–22 nt single-stranded RNA molecules that typically function as repressors of gene translation. While the precise mechanism by which they accomplish this end is still a topic of debate, our current understanding is that they bind to complementary sequences on mRNAs and act through a combination of translational inhibition and catalytic decay of their targets. They make up a significant part of the gene regulatory network (GRN) of all multicellular plants and animals and act in concert with transcription factors (TFs) to allow these organisms to perform complex regulatory functions.

Among these functions, miRNAs have been demonstrated to be of particular importance in cell differentiation and development. Knockout experiments in mice and zebrafish embryos have shown that disabling the entire miRNA regulatory system leads to early developmental failure [1–3]. However, despite their importance in early development, miRNAs may not be necessary for continued development following the establishment of certain cell types and basic morphological patterns [4,5]. These experiments suggest that miRNAs are an important source of robustness for developmental gene patterns and phenotypes in a broad sense. Consequently, a major focus of recent research has been on the ways in which miRNAs provide this robustness.

Three closely related hypotheses for the niche filled by miRNA-mediated regulation have been proposed in the literature. The first hypothesis is that miRNAs provide stability by reducing gene expression noise. Synthetic biology experiments, combined with mathematical models, have shown that miRNAs can function as a buffer of this noise, thereby improving the predictability of protein levels in the cell [6,7]. The second hypothesis is that miRNA circuits function to fine-tune gene expression and form multi-stable gene expression profiles. To support this hypothesis, it has been shown that in conjunction with TF-mediated feedback, these circuits can produce switch-like behaviour in gene expression levels and create stable alternative steady-state gene expression profiles [8–10].

The third hypothesis, which we investigate in this work, is that miRNAs are responsible for the regulation of cell differentiation *timing* and orchestration of tissue development. This role has been firmly established for some miRNAs, particularly in *C. elegans* [11,12]. In fact, the first identified miRNA, *lin-4*, was known to regulate developmental timing prior to the discovery that the gene product was not, in fact, a protein-based TF [13].

While these studies have provided multiple useful frameworks for modelling of miRNA–mRNA interactions and their potential robustness-conferring roles, they have primarily focused on small units of only one or a few TFs and miRNAs. However, a number of peculiarities of miRNA-mediated regulation at the whole-genome level suggest that the effects seen in these studies may not scale up in a way that explains the dependence of higher organisms on miRNAs. First, while multicellular organisms depend on the presence of miRNAs in general, experimental evidence shows that such organisms are quite robust with respect to perturbations of the miRNAome. For example, most knockouts of one or several miRNAs in *C. elegans* have been shown to produce no developmental or phenotypic abnormalities [14,15]. Similarly, humans heterozygous for the miRNA-processing enzymes *DICER1* and *DGCR8* carry an increased risk of certain paediatric tumours; however, most individuals are developmentally normal despite having miRNA levels which can vary by an order of magnitude or more [16–19]. Second, synthetic, theoretical and *in vivo* studies of transcription-only gene regulatory circuits have demonstrated that TFs are capable of sophisticated and precise control of gene expression noise [20] and also form robust systems with switch-like behaviour [21]. Taken together, these findings seem to obviate the specific need for miRNAs to perform these developmental tasks.

In this work, we investigate the plausibility of the proposed role of miRNAs as controllers of differentiation timing at the whole-genome level. We first create a theoretical framework which generalizes the concept of cell differentiation in an organism-independent sense. Within this framework, we use stochastic simulations to examine the effect that miRNAs have on differentiation timing. Our results show that miRNAs have a unique capability to act as retardants of differentiation, one that cannot be easily replicated by TFs alone. Because of the large number of unknowns in gene expression dynamics and gene regulatory network structure, we first demonstrate this role for miRNAs using the simplest possible assumptions for rate laws and network structures. We then validate our findings by showing that the results still hold under an alternative set of equations and assumptions, including those based on published studies specific to human gene regulation and the human genome itself. Importantly, these findings show how the dynamics of miRNA-mediated regulation at the molecular level can lead to the altered differentiation dynamics seen in *in vivo* studies. Additionally, our model demonstrates a novel application and implementation of a hybrid agent- and population-based simulation which can accommodate the wide-ranging scales of gene expression at the DNA, RNA and protein level. This model is capable of exactly simulating the expression dynamics of large, deeply connected GRNs and can help advance the development of whole-cell computational models.

## 2. Material and methods

Our general approach is to randomly generate GRNs and then evaluate the change in differentiation time when the set of active miRNAs changes. This presents two important challenges. First, how should one generate ‘acceptable’ GRNs? Second, what do we mean by ‘differentiation’? In this section, we address these questions and describe the stochastic model—additional information on these topics can be found in electronic supplementary material, S1 B.

### 2.1. Model theory

#### 2.1.1. GRN generation and analysis

While miRNAs have been found in unicellular organisms [22], they do not appear to be essential for survival. To the best of our knowledge, a tissue-based plant or animal which can function without any miRNA-mediated regulation has not been identified (see electronic supplementary material, table S1 for an overview). The diversity of miRNA-dependent organisms appears to imply that any phenotype-level general phenomena (such as developmental arrest) should be present independent of any particular GRN.

While it would be ideal to have well-studied ‘real-life’ GRNs for test organisms, it has recently been shown that current publicly available representations of even the thoroughly researched TF-only *E. coli* GRN are remarkably inconsistent with genomic data [23].

To accommodate this uncertainty, we generate networks using basic connectivity parameters rather than trying to infer which topological characteristics are most biologically relevant. It is critical to note that connectivity is only one property of GRNs and does not address the variety of important topological properties that are relevant to regulatory networks of all kinds. This is a key limitation of our GRN generation methodology, which we hope to improve as more consistent complex GRNs are resolved. Initially, we generate GRNs using parameters which are as simple as possible. For example, because the number of TFs (resp. miRNAs) that interact with a particular gene (resp. mRNA) is integer-valued, bounded and non-uniform, we generate GRNs in which indegrees are binomially distributed and meant to approximate the mean and median connectivities from a composite of six publicly available human gene regulatory databases (electronic supplementary material, figure S8A). Subsequently, we challenged our results by tailoring our model to more closely represent mammalian gene regulation; this is described in the main results. While the consistency of these datasets is uncertain, testing our model against an experimentally based set of degree distributions can provide some validation of our initial findings.

#### 2.1.2. Boundary conditions and time to differentiation

**Figure 1.**
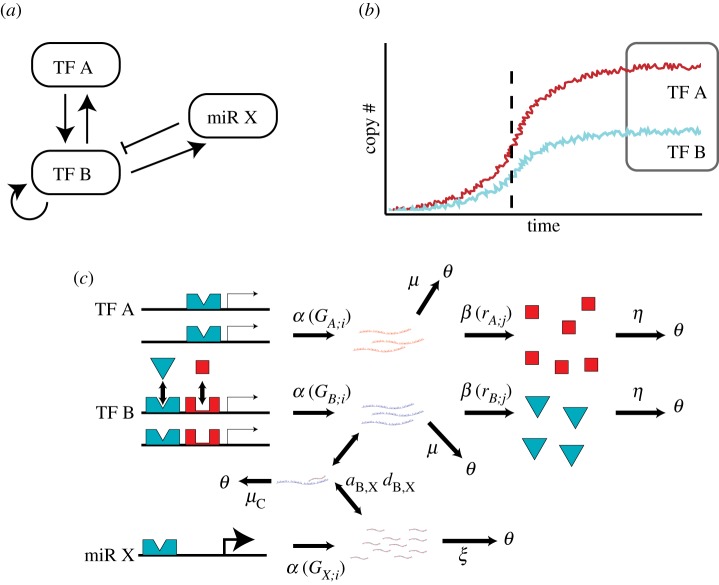
Illustrated representation of gene regulatory model. (*a*) A two-TF single-miRNA example network in ‘shorthand.’ TF effects (*arrows*) may be activating or inhibiting. (*b*) Sample TF expression trajectory. The cell begins in an undifferentiated state, the differentiated state corresponds to the final expression profile (grey box). The differentiation time corresponds to the point of maximal velocity (grey dashed line). (*c*) Detailed illustration of reactions in an example network. Note the subscripts of genes and mRNAs, denoted here with *i*, *j*—each molecule is tracked individually in the model with its own ‘binding state.’ Transcription and translation reaction propensities depend on this state. In this example, the transcription rate *α* is a function of the molecule states *G*_*A*;*i*_, *G*_*B*;*i*_, *G*_*X*,*i*_, where *i* refers to the *i*th molecule of the gene. Similarly, the translation rate *β* is a function of the mRNA states *r*_*A*;*j*_, *r*_*B*;*j*_. *μ*, *μ*_*C*_, *η*, *ξ* refer to the decay rates of the free transcript, bound transcript, TF and miRNA, respectively. (*a*, *d*) correspond to the association and dissociation rates of mRNA and miRNA; rates of TF-DNA association and dissociation omitted for space. (Online version in colour.)

Because our model is specifically focused on differentiation, we consider the GRNs in this study to be the restriction of a whole-genome GRN to the subnetwork consisting of lineage-specifying TFs and miRNAs. From this perspective, an undifferentiated (stem) cell corresponds to the zero state in which no copies of any mRNA or TF are present. Similarly, the differentiated state corresponds to a long-term stable TF expression profile. Since our model is stochastic, this does not refer to a single point in gene expression space, but rather to a certain region with low exit probability. Although we do examine the effect of miRNAs on long-term TF expression levels (see §§3.1.2), we make no assumptions about what the differentiated profile should look like in order to maintain an organism-agnostic perspective. Time to differentiation is the point in which TF expression has maximal ‘instantaneous velocity,’ obtained using the discrete Hilbert transform of the normalized TF expression trajectories (electronic supplementary material, S1). These concepts are outlined in figure 1.

Though it is a simplification, our representation of the undifferentiated cell is biologically reasonable. Because TFs are not exerting any feedback, all genes have a small constant probability of spontaneous transcription. It has been shown that low-level transcription of lineage-specifying genes is a defining feature of embryonic stem (ES) cells [24]. Additionally, it has been shown that ES cells have much lower transcript counts when compared to their differentiated counterparts: in ES cells, mRNA transcript counts are of the order of 10^4^ per cell, while in differentiated fibroblasts, transcript counts are approximately 20 times greater [25].

That said, it is important to acknowledge some key limitations of this simplification as well. Many stem cell-specific TFs and miRNAs have been identified and studied in detail, and their roles have been proven to be essential for stem cell maintenance and self-renewal [26]. Additionally, our model does not account for the cell cycle and cell division, both of which are fundamental to stem cell identity. To address this first issue, we examine the effect of alternative initial conditions on differentiation timing (§3.1.3); incorporation of the cell cycle into our gene expression model is planned as part of a future study.

### 2.2. Model equations and variables

In this section, we use the term ‘gene’ to refer to a segment of DNA encoding either a TF or miRNA. Let $\bar{G}$, $\bar{m}$, $\bar{r}$, $\bar{P}$ denote the number of species of genes, mRNAs, miRNAs and proteins, respectively. Because all mRNAs code for proteins, and all genes are either protein or miRNA-coding, we have $\bar{m}=\bar{P}$ and $\bar{G}=\bar{m}+\bar{r}$. We denote the set of these species by

$$\begin{array}{rl} G & =\{G_{1},G_{2}\ldots ,G_{\bar{G}}\}\\ R & =\{R_{1},R_{2}\ldots ,R_{\bar{G}}\}\\ & =m⊕r=\{m_{1},m_{2},\ldots ,m_{\bar{m}}|r_{1},r_{2},\ldots ,r_{\bar{r}}\}\\ \text{and}\;P & =\{P_{1},P_{2}\ldots ,P_{\bar{P}}\} \end{array}\;$$

When indexing, we use a semicolon to identify a particular molecule within a species: e.g. *G*_*i*;*j*_ refers to the *j*th molecule of gene *G*_*i*_. This is important, because we must keep track of the elements (TF or miRNA) bound to each molecule of DNA and mRNA in order to calculate associated reaction rates.

#### 2.2.1. Genes

Gene copy numbers do not change—i.e. the number of molecules of each *G*_*i*_ is immutable. Additionally,

(1) Each molecule of *G*_*i*_ has a basal transcription rate *α*_*i*_.
(2) For each gene species *G*_*i*_, there is a vector $p_{i}\in {\left\{0,1,2\ldots \right\}}^{\bar{P}}$, where (***p***_*i*_)_*j*_ corresponds to the number of binding sites for *P*_*j*_ to *G*_*i*_.
(3) For each *molecule* of *G*_*i*_, *G*_*i*; *k*_, there is an *occupancy vector* $o_{i;j}\in {\left\{0,1,2\ldots \right\}}^{\bar{P}}$, where each (***o***_*i*;*j*_)_*k*_ ≤ (***p***_*i*_)_*k*_.
(4) For each (*G*_*i*_, *P*_*j*_) binding pair, there is a transcriptional modifier *λ*_*ij*_ which corresponds to the effect of *P*_*j*_ on the transcription rate on the molecule of *G*_*i*_ it is bound to.
(5) Each *λ*_*ij*_ ∈ (0, ∞). For activating transcriptional effectors, *λ*_*ij*_ > 1. For inhibitory effectors, *λ*_*ij*_ < 1.
(6) For each (*G*_*i*_, *P*_*j*_) binding pair, association and dissociation kinetics are first-order and are denoted by *γ*_*ij*_ and *δ*_*ij*_, respectively.

#### 2.2.2. mRNA

mRNA species behave quite similarly to genes with two exceptions: mRNAs can be created and destroyed, and miRNAs can accelerate mRNA decay and reduce translation rates. Additionally,

(1) Each molecule of *m*_*i*_ has a basal translation rate *β* and a basal decay rate *μ*_0_.
(2) For each mRNA species *m*_*i*_, there is a vector $q_{i}\in {\left\{0,1,2\ldots \right\}}^{\bar{r}}$, where (***q***_*i*_)_*j*_ corresponds to the number of binding sites for *r*_*j*_ to *m*_*i*_.
(3) For each *molecule* of *m*_*i*_, *m*_*i*; *k*_, there is an occupancy vector $\omega_{i}\in {\left\{0,1,2\ldots \right\}}^{\bar{r}}$, where (****ω****_*i*;*j*_)_*k*_ ≤ (***q****i*)_*k*_.
(4) For each (*m*_*i*_, *r*_*j*_) binding pair, there is a translational modifier *κ*_*ij*_ which corresponds to the effect of *r*_*j*_ on the transcription rate on the molecule of *m*_*i*_ it is bound to.
(5) For each (*m*_*i*_, *r*_*j*_) binding pair, there is a decay modifier *μ*_*ij*_ which corresponds to the effect of *r*_*j*_ on the decay rate on the molecule of *m*_*i*_ it is bound to.
(6) miRNAs can only decrease translation rates and increase decay rates of their bound targets—i.e. all *κ* ∈ (0, 1) and all *μ* ∈ (1, ∞).

#### 2.2.3. TF and miRNA

Unlike the previous two molecule types, our model treats free TFs and miRNAs as homogeneous populations, so we do not need to keep track of the state of each molecule. The binding of free TF and free miRNA to their targets follows mass-action laws:

1. A molecule of free *P*_*j*_ decays with rate *η*_*j*_, while a molecule of free *r*_*j*_ decays with rate *ξ*_*j*_.
2. A molecule of free *P*_*j*_ binds to and dissociates from an available site on *G*_*i*_ with propensities *γ*_*ij*_, *δ*_*ij*_. The corresponding coefficients for a molecule of free miRNA *r*_*j*_ binding to and dissociating from mRNA *m*_*i*_ are denoted *a*_*ij*_, *d*_*ij*_, respectively.

These rules and reactions are listed in table 1 and are illustrated for a single gene/mRNA species in electronic supplementary material, figure S2.

**Table 1.** Reactions for stochastic simulation for a *single molecule*: the *i* (species id) and *k* (molecule id) subscripts are suppressed. It is assumed that *G*_*i*_ → *R*_*i*_ → *P*_*i*_ (last reaction if *G*_*i*_ is TF-coding). Note alternative stochiometries and propensities for transcription, translation and mRNA decay.

name	stoichiometry	propensity
association (*P*_*j*_)	$G(o)+P_{\;j}\to G(o+1_{\;j})$	*γ*_*j*_ (***p***_*j*_ − ***o***_*j*_)|*P*_*j*_|
dissociation (*P*_*j*_)	$G(o)\to G(o-1_{\;j})+P_{\;j}$	*δ*_*j*_*o*_*j*_
association (*r*_*j*_)	$m(\omega )+r_{\;j}\to m(\omega +1_{\;j})$	$a_{\;j}(q_{\;j}-\omega_{\;j})|r_{\;j}|$
dissociation (*r*_*j*_)	$m(\omega )\to m(\omega -1_{\;j})+r_{\;j}$	*d*_*j*_*ω*_*j*_
miRNA decay	*r*_*j*_ → 0	$\xi_{\;j}|r_{\;j}|$
TF decay	*P*_*j*_ → 0	$\eta_{\;j}|P_{\;j}|$
**Model 1** (simplified)		
transcription	G(o)→G(o)+R	$\alpha \prod_{l=1}^{\bar{P}}\lambda_{l}^{o_{l}}$
translation	m(ω)→m(ω)+P,	$\beta \prod_{l=1}^{\bar{P}}\kappa^{\omega_{l}}$
mRNA decay	m(ω)→∅	*μ*_0_
**Model 2** (extended)		
transcription	*G* → *G* + *R*,	$\alpha \frac{\prod_{l=1}^{\bar{P}}\lambda_{l}^{o_{l}}}{K_{C}+\prod_{l=1}^{\bar{P}}\lambda_{l}^{o_{l}}}$
translation	m(ω)→m(ω)+P,	$\beta \frac{\prod_{l=1}^{\bar{P}}\kappa^{\omega_{l}}}{K_{L}+\prod_{l=1}^{\bar{P}}\kappa^{\omega_{l}}}$
mRNA decay	$m(\omega )\to \left\{ \begin{array}{cc} \emptyset ,\; & \mathrm{no}\;\mathrm{recycling}\;\\ \sum_{\;j}\omega_{\;j}r_{\;j}, & \mathrm{with}\;\mathrm{recycling} \end{array}\right.$	$\mu_{0}\frac{\prod_{l=1}^{\bar{P}}\mu^{\omega_{l}}}{K_{L}+\prod_{l=1}^{\bar{P}}\mu^{\omega_{l}}}$

When an association reaction takes place, e.g. between *G*_*i*_ and *P*_*j*_, we choose a random starting molecule of *G*_*i*_ and walk through each molecule until an available binding site is found. The exact same process takes place for dissociation reactions, looking for a randomly selected molecule which has a *P*_*j*_ bound to it.

Unlike the more intuitive association, dissociation and miRNA/TF decay reactions presented, the dynamics of transcription, translation and mRNA decay in the presence of miRNAs are still a subject of much debate. For example, it is known that miRNAs can act either by direct inhibition of translation or by enzymatic degradation of their targets [27,28] (reviewed in [29]). Similarly, the effect of TFs on target genes consists of multiple potential mechanisms which differ depending on the context [30,31].

To address these uncertainties, we use two model representations to derive our results. The first model uses the simplest possible rate laws and assumes all *μ*_*ij*_ = 1, while the second model uses an alternative set of rules. This model decreases the number of overall parameters in order to identify whether miRNAs can alter differentiation time. We then challenge these results using an ‘extended’ model with dynamics that are based on previously published experiments and are also meant to address ongoing debates in the field of miRNA dynamics. This challenge is meant to validate the results obtained using the first model to show that the qualitative effects observed are not tied directly to one or the other side of any of these debates. The alternative rules are described in §3.2.

### 2.3. Model parameters and simulation process

Our simulation timescales and parameters are measured in hours, and simulations were run for a total of 5000 simulated hours each. Default parameters can be found in electronic supplementary material, tables S2 and S3; number of networks tested are listed in electronic supplementary material, tables S4 and S5.

## 3. Results

### 3.1. Base model

#### 3.1.1. miRNAs delay time to differentiation

**Figure 2.**
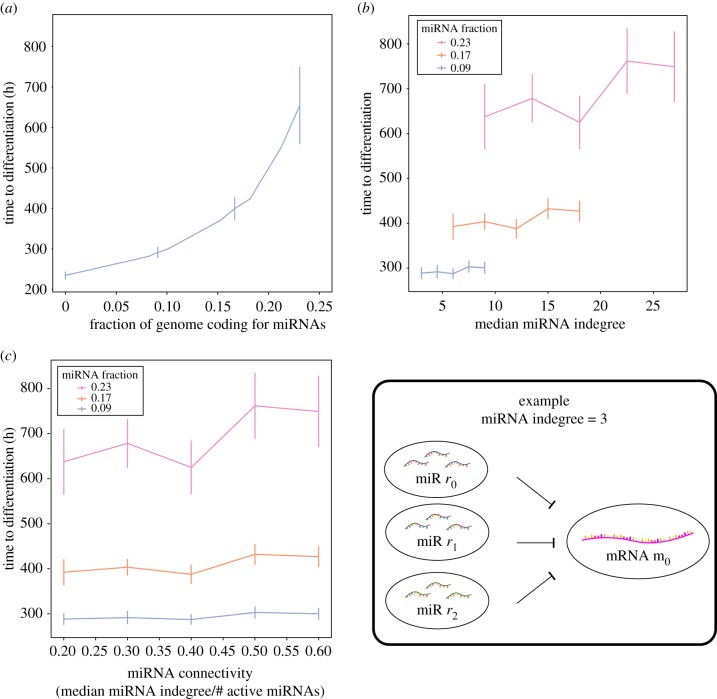
Time to differentiation increases with the fraction of the genome encoding miRNAs. (*a,b*) The relationship appears to depend primarily on the fraction of genome encoding miRNA/colon networks with the same median miRNA indegree but fewer total miRNAs have distinctly shorter times to differentiation. (*c*) While the fraction of miRNAs in the genome has a large effect on differentiation time, the relative connectivity within the GRN has a more limited effect across a wide range of regulatory network structures. miRNA indegree corresponds to the number of miRNA species that can target a particular mRNA species (lower right). (Online version in colour.)

Almost universally (in ≈99% of trials), the addition of miRNAs to GRNs increases time to differentiation (figure 2*a*), and this effect appears to be independent of genome size (electronic supplementary material, figure S3A). Rather, the effect appears to depend primarily on the ratio of the size of the activated miRNA subset to the total genome size, which we call the ‘miRNA fraction’: (#miRs)/(#miRs+#TFs). Furthermore, the degree of miRNA–mRNA connectivity did not produce a significant change in absolute differentiation time (figure 2*b*,*c*), suggesting that on the individual gene level, whether an mRNA is or is not a target of miRNAs is more important than the precise number (or multiplicity) of miRNAs targeting it. This relationship between differentiation time and miRNA fraction is remarkably robust: with respect to differentiation time, increasing the number of miRNAs targeting a particular gene does not appear to compensate for a reduction in miRNA fraction.

Experimental studies in both mice and zebrafish have shown that the presence of miRNAs appears to sustain the embryonic stem (ES) cell phenotype on a timescale of the order of 10^2^ h [3]. While the number of miRNAs in any genome is unknown, common estimates are of the order of 10^3^ in these organisms, which would correspond to approximately 10% of expressed genes in their corresponding ES cells [32,33]. For miRNA fractions in this range, we found that the delay was in the range of about one week, consistent with these studies.

#### 3.1.2. Effect on protein production

**Figure 3.**
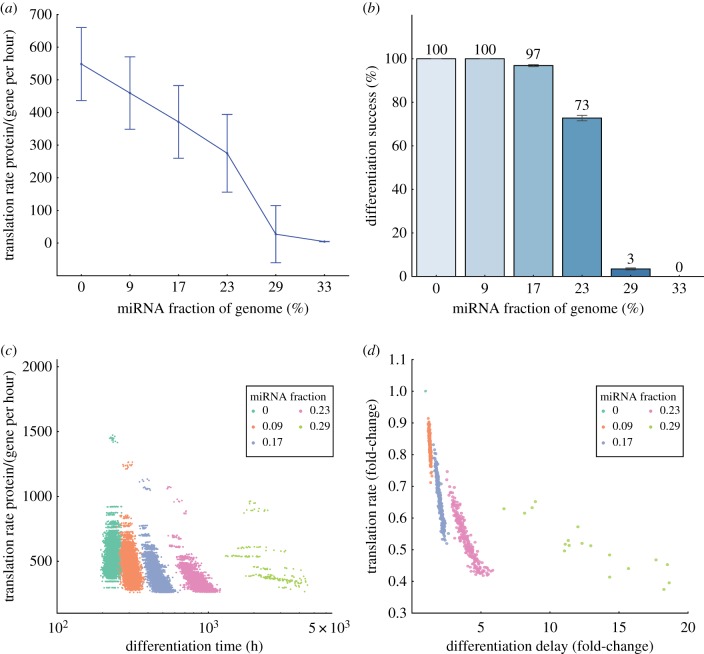
Increased miRNA content in the genome reduces translation rate. (*a*) As miRNA fraction increased up to approximately 23%, the relationship was nearly linear with an approximately 4.5% reduction in average TF copy number per miRNA in the differentiated state. However, additional miRNAs beyond this level had a much more dramatic effect on protein translation. (*b*) Differentiation success rates. miRNAs at levels below 23% typically were successful in differentiation; beyond that level, differentiation fell off rapidly. (*c*) Addition of miRNAs couples differentiation time and TF translation rates in successful differentiation events (normalized per-network in (*d*)). Protein output increase by miRNAs is limited to a roughly fourfold increase (translation rate × delay) when compared to the GRN without any miRNAs present. Note that all trials with a miRNA fraction of 0.33 resulted in differentiation failures and are not shown. (Online version in colour.)

In line with the role of miRNAs as repressors of translation, we observed that TF synthesis rates generally decreased as miRNAs were added to the network as a result of a reduction in translation rates (figure 3*a*). As one might expect, miRNAs remove transcribable mRNAs from the RNA pool, thereby decreasing long-term TF copy numbers. For a range of miRNA levels, miRNA quantity had a near-linear effect on TF copy numbers. Interestingly, this reduction amounted to a roughly 4.5% decrease in TF copy number per added miRNA, in agreement with previous experimental estimates of a 3.5–5.6% reduction in protein copy number per miRNA [34].

At the highest levels, however, miRNAs appeared to decrease protein translation rates to a point of apparent breakdown, in which differentiation fails to take place. As an indicator of differentiation ‘success,’ we examined whether post-differentiation translation rates increased beyond basal levels (200 TFs per gene per hour, the rate of translation with no TF or miRNA participation at all). We observed that at these lower levels (up to a miRNA fraction of roughly 23%), miRNAs had minimal, if any, effect on actual differentiation success (figure 3*b*). In line with our observations for differentiation time and translation rates, success rates dropped precipitously when miRNA content exceeded these levels. This thresholding effect suggests that rather than exhibiting universal control over differentiation time, oversaturation of the genome/transcriptome with miRNAs leads to a permanent failure to differentiate.

The notion that excessive miRNA content may result in a failure to differentiate led us to ask whether miRNAs created an upper bound on the amount of TF produced by undifferentiated cells. Such a barrier would ensure that the total protein output of the undifferentiated cell would be limited, acting as a fail-safe to prevent the indefinite proliferation of stem cells. Indeed, we found that the addition of miRNAs to the genome created an interdependence between mean translation rates and differentiation time (figure 3*c*). When normalized on a per-network basis (figure 3*d*), we saw that miRNAs did create an interdependence of translation rates and differentiation times. Using total protein output as a (very coarse) surrogate for overall proliferation, our results indicate that miRNA-mediated regulation might be able to increase the total stem cell output by approximately two- to fourfold prior to differentiation.

#### 3.1.3. Transient stabilization is unidirectional

Functional TF copy numbers in mammalian cells have been shown to span a wide range from 0 molecules up to 10^6^ or more [35,36]. These numbers stand in stark contrast to their target genes, whose copy numbers are typically small and fixed. Therefore, the effect of many TFs likely saturates at some intermediate concentration, while miRNAs, whose efficacy depends upon target mRNA levels, would diminish as the level of target mRNAs increases regardless of miRNA quantity [37]. Since it appears that miRNAs can delay the onset of TF-mediated feedback, we presumed that the capability of miRNAs to delay differentiation was sensitive to initial TF copy number. In particular, we wanted to determine whether the differentiation time delay was observed when initial TF copy numbers were above steady-state levels.

**Figure 4.**
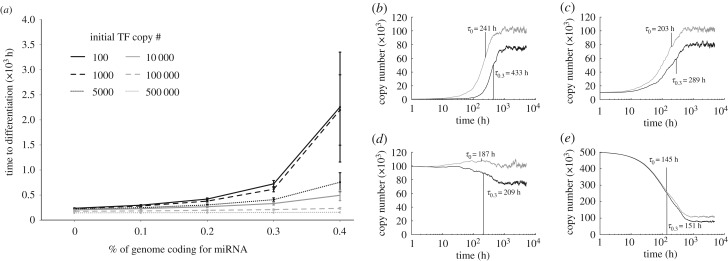
MicroRNAs disproportionately affect low copy number genes. (*a*) The ability of miRNAs to delay differentiation time depends strongly on ambient TF levels, with a strong fall off in delay efficacy at roughly 5 × 10^3^ copies (dotted, black). (*b*–*e*) Sample traces of a single TF trajectory with miRNAs absent (grey) and with a miRNA fraction of 0.3 (black) for initial TF copy numbers of 10^2^ (*b*), 10^4^ (*c*), 10^5^ (*d*), and 5 × 10^5^ (*e*). Times to differentiation both with and without miRNAs present (τ_0_,τ_0:3_) converge towards TF decay rate (10^−2^/h) as TF quantities increase.

Under a range of initial conditions, we found that the delay effects of miRNAs are only observed when TF copy numbers approached steady-state from below, and not above (figure 4). When initial TF counts exceeded typical steady-state values, the equilibration process became limited by the rate of protein decay, which was of the order of 10^−2^/h. Importantly, this change appeared to occur when initial TF copy numbers crossed a level of approximately 5000 molecules per TF per cell (figure 4*c*,*d*), in agreement with experimentally observed TF copy numbers during the course of differentiation [36].

### 3.2. Sensitivity to unknown parameters

One of the major issues with modelling both TF-DNA and miRNA–mRNA interactions is the limitations in our current knowledge of the kinetics and dynamics of these interactions. Additionally, there are a number of major topics under debate with respect to miRNA-mediated regulation. To understand how these open questions may affect our findings, we evaluated whether our results held true using an extended, mammal-specific model using alternative transcription, translation and mRNA decay dynamics (table 1).

In conjunction with these tests, we tailored our GRN generation to currently available gene regulatory data. Specifically, we used maximum-likelihood estimation on our composite human gene regulatory data to fit a displaced, clamped negative binomial distribution (electronic supplementary material, figure S8B).

#### 3.2.1. Transcriptional and translational dynamics

The modelling and analysis of transcription and translation are topics that have been studied in great detail, revealing a wide variety of mechanisms and kinetic equations for each (e.g. [38,39]). In the previous sections, we used a linear model for transcriptional and translational propensity, where transcription and translation rates were proportional to the product of the independent effects of all bound TFs (transcription) or miRNAs (translation). This behaviour was chosen for its simplicity, as well as for its ability to accommodate a high dynamic range of transcription/translation rates (as observed in [30,36]), whose limits are set strictly by the number of binding sites on either a gene’s promoter region or the mRNA itself.

However, a number of different models have been tested and are applicable in different contexts. While we cannot test them all, we wanted to see if the choice of an alternative transcriptional and translational rate law would reproduce the delay effects previously observed. The Hill-type kinetic model is commonly used for modelling transcriptional ([31,40]) and translational regulation and has been extensively applied to the study of miRNA-mediated circuits [6,41,42]. Furthermore, a direct comparison of multiple proposed rate laws found that the Hill-type equation was the best fit for a number of RNA interference datasets in mammalian cells [28]. In our model, the number of binding sites (Hill coefficient) is already part of our model; the new rate laws are shown in table 1.

**Figure 5.**
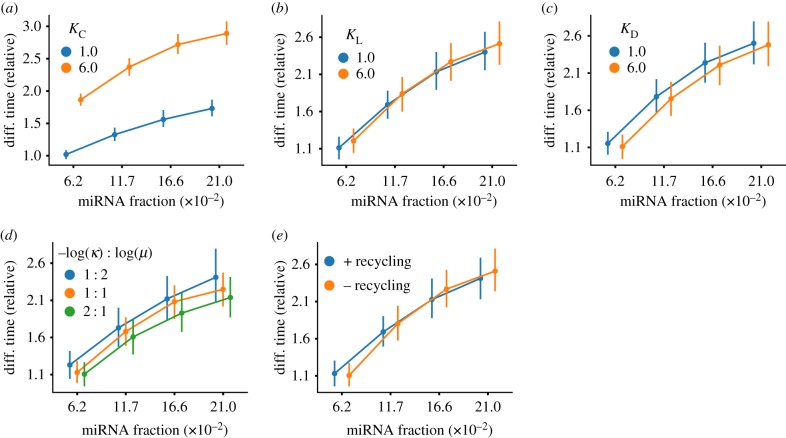
Variations in transcriptional and translational kinetics and dynamics. (*a*) Variation of the Michaelis–Menten constant *K*_C_ can have a significant impact on differentiation delay; reducing the significance of TF-mediated control can nearly extinguish the observed delay. However, changing the constants corresponding to protein translation *K*_L_ (*b*) and mRNA decay *K*_D_ (*c*) do not have the same effect. (*d*) Modifying the relative contributions translation inhibition versus catalytic degradation by miRNAs does not appear to alter differentiation delay dynamics. Similarly, the effect of miRNA recycling after miRNA-mediated decay does not significantly alter delay effects (*e*). In (*a–c*), ranges for constants were tested in a range from 10^−2^ to 10^2^ (see electronic supplementary material, table S5). The delay patterns remained the same, so for readability, only two intermediate values are shown. (Online version in colour.)

To test the extended model, we varied each of *K*_C_, *K*_L_, and *K*_D_ across a range from 10^−1^ up to 10^2^ for 27 648 total GRNs (electronic supplementary material, table S5). Our results showed that under these alternative assumptions, miRNAs maintained their differentiation-delaying effect (figure 5). Importantly, the effect was only diminished when the Michaelis–Menten-like constant *K*_C_ was decreased (figure 5*a*) and appeared to be insensitive to modest changes in the translational and mRNA decay constants (figure 5*b*,*c*). Reducing *K*_C_ weakens the effect of activating TFs on transcription rates by decreasing the transition threshold from first-order to zero-order kinetics, indicating that TF-mediated feedback is an important contributor to the observed effects of miRNA-mediated inhibition. By comparison, since translational regulation by miRNAs is strictly inhibitory, their relative effect on translation rate is less sensitive to changes in this constant.

#### 3.2.2. Catalytic decay, sequestration and miRNA recycling

There is an ongoing debate about the precise effect of miRNAs on their targets. In plants, it is believed that miRNAs act primarily by cleaving their target mRNAs, reducing the pool of total mRNA, while in animals, it is believed to be some combination of cleavage and direct translational inhibition (mRNA sequestration) [43–45]. Naturally, the primary difference between these two modes of action is that the latter is a reversible process, while the former is irreversible. In *in vitro* experiments, experimental data appeared to fit well to mathematical models which assumed either sequestration [6] or degradation [27] to be the dominant mechanism of action.

Similar to the model of [6], we have thus far assumed that sequestration is the primary mechanism of miRNA-mediated translational inhibition. However, given the ongoing debate around this topic, we wanted to see if our results persisted regardless of the main mode of action of miRNAs on their targets. We incorporated this catalytic decay through the additional reaction in table 1 (Model 2) and examined how varying the sequestration-to-degradation ratio affected differentiation time.

Varying of the ratio of these effects did not significantly alter the delay dynamics (figure 5*d*), although a slight decrease in relative differentiation time when catalytic degradation was heavily favoured. The finding that the two mechanisms may result in similar outcomes generally agrees with the detailed analyses of different mechanisms found in [39,42,46].

Because many of the aforementioned studies focused on small networks, a related, but separate question largely remains unanswered: if an mRNA binds multiple miRNAs, what happens to those miRNAs following the degradation (catalysed or spontaneous) of their target? It has been proposed that whether miRNAs are ‘recycled’ or not does not have a profound effect on overall dynamics [47]. Nonetheless, it has been shown experimentally that single miRNAs can regulate several targets during their lifetime, suggesting that some recycling likely takes place [44,48]. In our model, the effects on time-to-differentiation were virtually indistinguishable whether miRNAs were completely recycled or completely destroyed following target degradation (figure 5*e*). This likely comes from the fact that in cell cultures, actively transcribed miRNAs typically outnumber their mRNA targets by one to three orders of magnitude in steady-state conditions [44]; therefore, the contribution of the recycled miRNAs to the free miRNA pool is small.

#### 3.2.3. TF effect direction and magnitude

In addition to the debate over the rate laws governing transcriptional rates, little is known about the range of magnitudes of TF effects on their targets. Recent research has shown that TF effects are context-dependent and can range from complete transcriptional repression to a several hundred-fold increase in transcription rates [30]. While the true dynamics of TF-mediated regulation are certainly much more complex, a number of models have drawn TF effect sizes from a normal distribution [49–51] and used the signedness of the effect size to distinguish activation from inhibition. In these models, TFs accumulate additively. By contrast, because the effect of TFs in our model is cooperative, the closest analogue of this is the selection of TFs from a lognormal distribution, where effect sizes less than one correspond to repression and effect sizes greater than one correspond to activation.

**Figure 6.**
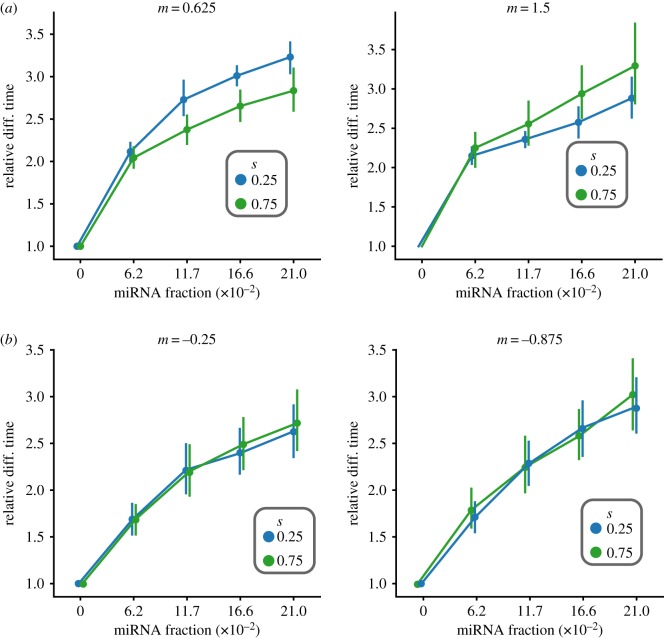
Relative differentiation time using lognormally distributed TF effect sizes with log mean and standard deviation indicated by *m*, *s*. The miRNA-mediated differentiation delay persists whether the average TF effect $\bar{\lambda }$ is activating (*a*) or inhibiting (*b*). (Online version in colour.)

We found that the use of lognormally distributed TF effect sizes did not change the pattern of miRNA-mediated differentiation delay (figure 6). Importantly, a consistent delay pattern was observed regardless of whether the mean TF effect was activating (figure 6*a*) or inhibiting (figure 6*b*). This is important, as miRNAs are known to be critical regulators of heterochronic genes which are only transiently activated and later repressed [11,52]; therefore, miRNAs can exert their effect when genes transition from activating to inhibiting modes and vice-versa.

### 3.3. Does stochasticity matter?

As previously mentioned, mathematical modelling and synthetic biology studies have demonstrated that miRNAs are important buffers of stochastic transcriptional noise. At first glance, our findings suggest that miRNAs could cause a delay in differentiation in a similar way. In our model, miRNAs can serve to mitigate unwanted, random transcriptional events, thereby reducing stochastic fluctuations which could otherwise result in premature, deleterious differentiation outcomes.

However, our choice to use a stochastic model is based on the low copy numbers of genes and mRNAs. With only two copies of each coding gene, for example, the continuity of an ordinary differential equation (ODE) based model becomes a poor approximation. Stochastic approximations from ODE systems, such as the frequently used linear noise approximation, become inaccurate for small molecule counts, motivating our use of a discrete model [53]. In the electronic supplementry material, appendix, we demonstrate that for an ODE model of a single-TF single-miRNA system with positive feedback similar to that in [9], the delay effect remains in the absence of stochastic noise. Moreover, the ODE model also demonstrates the same potential for miRNA oversaturation—as the miRNA effect size increases, the system becomes bistable with a steady state at 0, similar to our observations (see electronic supplementary material, S1 A and example in figure S1).

## 4. Discussion

This study advances the field of miRNA research in two important ways.

### 4.1. Understanding transient behaviour

Unlike previous studies, we specifically examine non-equilibrium behaviour. This behaviour is important, because embryonic development is a fundamentally transient process and requires orchestration. It is not enough for the genomes of tissue-based organisms to have stable states corresponding to the desired tissue types—the appearance of such tissues during development has to be carefully timed to meet the needs of the developing embryo. Therefore, understanding the transient behaviour of gene expression is especially important.

miRNA-mediated regulation extends well beyond zygotic- and germ-layer developmental stages and has been shown to be essential for the development of a wide range of tissues and organ systems, including haematopoiesis, brain and cardiac development and germ cell formation (electronic supplementary material, table S1). Despite this diverse range of developmental contexts, there is a common theme to many of the observed consequences of global miRNA suppression: namely, the formation of uncharacteristically small, often poorly patterned target tissues [54,55]. Similar experiments have typically shown that the potential physiological consequences of such malformations correlate with the timing of miRNA suppression induction, with more severe phenotypes associated with earlier induction times [3,4,56]. While these experiments conclude that miRNAs are essential for the proper development and morphogenesis of particular organisms and/or tissues, they do not examine the root cause of these defects.

Our findings can help explain such experimental results. By delaying cell differentiation, we propose that miRNAs give differentiating cells time to proliferate, and begin forming critical patterns and gradients required for cell specification and morphogenesis. Rather than destabilizing phenotypes, as has been previously suggested, our findings support the hypothesis that such abnormalities come from *premature* differentiation rather than *abnormal* differentiation of stem, precursor and progenitor cells. Based on our findings, we believe that while this effect generally depends on the proportion of active miRNAs in the genome, it is largely independent of particular network topologies or individual miRNA sequences themselves. In line with this theory, we predict that it should be possible to overcome the effects of disabling miRNA biogenesis by supplementation with any combination of exogenous miRNAs that span a similar range of targets to the host organism. For example, we propose that it may be possible to support a miRNA-less embryo through development by supplementation with a purified mix of miRNAs from mature, adult tissues, possibly from a different species altogether. In the same way, supplementation of miRNA-competent embryos with exogenous miRNAs may help to stabilize them and increase their lifetimes/viabilities.

### 4.2. Simulating genome-scale GRNs

Second, our mathematical model and simulation use a hybrid agent- and population-based approach to accommodate the differing scales of gene expression at the DNA, RNA and protein level. While such a model has been previously described in the literature [57], in this work, we demonstrate the utility in its application to simulating gene regulation. This implementation is effective because of the wide-ranging scales of molecular counts and complexity at the DNA, RNA and protein levels.

Each gene is encoded in the genome with a small, typically fixed copy number (e.g. 2). For an actively transcribed gene, only tens to hundreds of mRNA molecules are present in many cells. This small number of mRNAs can lead to the production of millions of protein molecules. While molecular counts increase substantially at each of these levels, configuration complexity follows an opposing trend. Gene enhancer regions can bind TFs in a tremendous number of different configurations with differing effects, which are essential to controlling transcriptional output [30]. Although multiple miRNAs can bind to a single mRNA in a variety of combinations as well, the fact that the effect of miRNAs appears to generally be unidirectional with a common mechanism of action across different miRNAs suggests that the effect of these combinations on translation rate has a narrower dynamic range. In fact, research suggests that the number of biologically significant interactions is actually much smaller than the total number of potential binding partners [58,59]. Consequently, our hybrid approach is well suited to accommodate these differing scales in which some entities have low molecule counts with many configurations while others have high molecular counts with fewer configurations.

In our model, we treat each gene and mRNA transcript as an individual entity with its own state, while each miRNA and/or TF molecule is treated as a homogeneous population. No averaging or approximation is used to represent the combination of bound TFs or miRNAs to a particular gene or transcript. Unlike other simulation tools, it is specifically geared towards large scale, highly interconnected networks with ‘stereotypical’ rate equations. Benchmarking and comparison to other implementations will be part of a future study, as will testing against bacterial and yeast GRNs. Source and documentation can be found at https://github.com/adpposner/GRNsim.

Undoubtedly, our work has only generated a hypothesis and merely provides a starting point for experimental studies to rigorously test and/or clarify. However, our results point to an important broader role for mathematical and computational modelling of biological systems. By abstracting the notions of undifferentiated cells, gene regulatory networks and regulatory processes, such modelling allows us to find and investigate the commonalities among different organisms and tissue types, without absolute knowledge of the intricate molecular details of complex gene regulatory mechanisms. By contrast, examining such theories in just a single organism or a single-cell type in a traditional wet laboratory environment requires specific, and often extensive, resources, skills and knowledge. In addition to providing a novel theory to explain the evolutionary integration of miRNAs in general, we hope this and other studies help to reaffirm the importance of theoretical modelling and simulation as valuable complementary approaches to classical wet laboratory experimentation in advancing our understanding of biological systems.

## Supplementary Material

Supplementary Methods and Appendices

## Data accessibility

Implementation information available in electronic supplementary material and all source code available at https://github.com/adpposner/GRNsim.
