# Supplementary material for: Connecting the molecular function of microRNAs to cell differentiation dynamics
Source: J R Soc Interface. 2019 Sep 25;16(158):20190437. doi: 10.1098/rsif.2019.0437 (PMC6769318; doi:10.1098/rsif.2019.0437)
Supplement: Supplementary Methods and Appendices [file rsif20190437supp1.pdf]

Supporting information

A Analytical basis

An important feature of this model system is that it does not directly depend on the stochasticity of gene expression. To see this, we consider a single lineage-specifying TF which is self-activating, and a separate miRNA which regulates it in the following system of equations similar to those from [1] and [2]. Here, we use R to denote the TF's mRNA, S to denote the miRNA, and P to denote the mature TF. In this representation, if the miRNA is capable of *catalytic* cleavage, we would have $\alpha < \lambda$; if not, we have $\alpha = \lambda$.

$$\frac{dR}{dt} = \frac{k_R P}{K_M + P} - \delta_R R - \lambda R S \tag{1}$$

$$\frac{dS}{dt} = k_S - \delta_S S - \alpha R S \tag{2}$$

$$\frac{dP}{dt} = k_P R - \delta_P P \tag{3}$$

It has been demonstrated experimentally that miRNAs must be present *prior to* the beginning of differentiation, and outnumber their targets by several orders of magnitude [3–5]. We use these facts to make a quasi-steady-state assumption on S, namely

$$S \approx \frac{k_S}{\alpha R + \delta_S} \tag{4}$$

Substituting equation 4 into equation 1:

$$\frac{dR}{dt} = \frac{k_R P}{K_M + P} - \delta_R R - \lambda \frac{k_S}{\alpha} \frac{R}{\frac{\delta_S}{\alpha} + R} \tag{5}$$

As expected this reduces to Michaelis-Menten kinetics and redefine λ, δ_S to simplify the system to:

$$\frac{dR}{dt} = \frac{k_R P}{K_M + P} - \delta_R R - \lambda \frac{R}{\delta_S + R} \tag{6}$$

$$\frac{dP}{dt} = k_P R - \delta_P P \tag{7}$$

Analogous to our model framework, we use the following initial conditions, where ϵ is assumed to be small:

$$R(0) = \epsilon, \; P(0) = 0 \tag{8}$$

Solving the first several terms of this initial value problem near $t = 0$ using a series expansion gives us

$$P(t) = \epsilon k_P t \left(1 - t \frac{\lambda + k_P (\delta_P + \delta_R)}{2(\delta_S + \epsilon)}\right) \tag{9}$$

$$+ \frac{k_P \epsilon t^3}{6} \left(\frac{k_R k_P^2}{K_M} + \delta_P^2 + \delta_R \delta_P + \delta_R^2 + \lambda \frac{\delta_P + \delta_R}{\delta_S + \epsilon} + \lambda \frac{\delta_S \delta_R}{(\delta_S + \epsilon)^2} + \lambda^2 \frac{\delta_S}{(\delta_S + \epsilon)^3}\right) \tag{10}$$

Denote the solution for P when $\lambda = x$ as P_x. The difference between protein levels with and without miRNA interference simplifies to:

$$P_0 - P_\lambda = \lambda t^2 \epsilon k_P \left(\frac{1}{2(\delta_S + \epsilon)} - \frac{t}{6}\left(\frac{\delta_P + \delta_R}{\delta_S + \epsilon} - \frac{\delta_S \delta_R}{(\delta_S + \epsilon)^2} + \lambda \frac{\delta_S}{(\delta_S + \epsilon)^3}\right)\right) \tag{11}$$

Looking at the lowest-order term, we see that it is maximized when $\lambda k_P \gg \delta_S$. It is clear from equation 11 that if we maximize $P_0 - P_\lambda$ in such a way that does not drastically alter the steady state value of P, then a delay effect identical to the main results will be achieved.

The steady-state value of P comes directly from the roots of:

$$k_P\delta_R\bar{P}^2 + (K_M\delta_P\delta_R + k_P\delta_R\delta_S + k_P\lambda - k_Pk_R)\,\bar{P} + K_M\delta_R\delta_P\delta_S + K_M\lambda\delta_P - k_Pk_R\delta_S = 0 \tag{12}$$

From this equation, we can see that λ only appears in two terms. To minimize the effect on $\bar{P}$, we want λ small compared to δ_R, while minimizing the contribution from the second term is equivalent to minimizing $\frac{K_M\delta_P}{k_P\delta_R}$. In addition to the same relationship between λ and δ_R, this part is minimized when the equilibrium constant $\frac{k_P}{\delta_P}$ is large, particularly when compared to K_M. Also worth noting is the fact that as λ increases, particularly when δ_S remains low, the system becomes stable at both 0 and a non-zero equilibrium. From a biological perspective, these criteria are quite reasonable. $\lambda < \delta_R$ implies that at steady state, most mRNA degradation takes place spontaneously, without miRNA-mediated acceleration, similar to, e.g. [6]. The criterion $\frac{k_P}{\delta_P} \gg 1$ is biologically equivalent to the notion that translation has a high gain, as noted in, e.g. [7].

The first criterion is that $\lambda \gg \delta_S$ also is reasonable. Referring to the original definition in equation 5, we are actually concerned with the ratio of the original λk_S to the original δ_S. As in the previous case, this corresponds to there being a high concentration of the miRNA S at equilibrium. This is also a well-known property, as in [5, 6]. While this ODE system is *not* genome-scale, it does demonstrate how the differentiation delay induced by miRNAs may not be a function of the stochastic nature of gene expression. This is important, because it implies that our findings are not directly related to the well-studied proposed stochastic noise-buffering behavior of miRNAs (see, e.g. [1, 8, 9]). An example of the numerical solutions to this type of system are given in Figure S1.

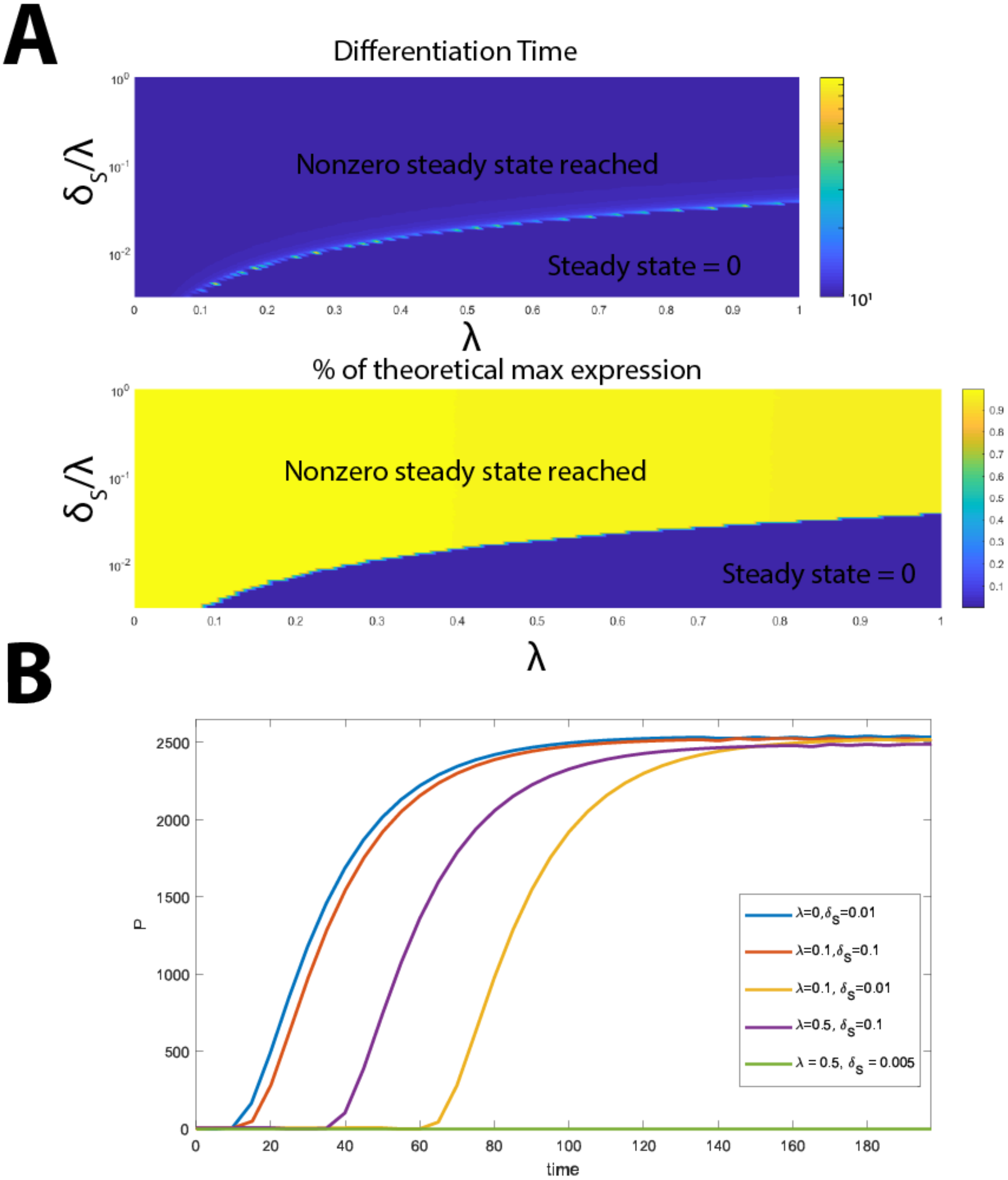

Fig S1. Example plots of single-TF single-miRNA approximation demonstrating delay effect. **A**) Upper, colormap of differentiation time showing delay effect and boundary of region which fails to differentiate. Lower, colormap of total percentage of max expression obtained. **B**) Plot of differentiation trajectory for sample λ, δ_S values. Note example of failure to differentiate for $\lambda = 0.5$, $\delta_S = 0.005$ ($\frac{\delta_S}{\lambda} = 10^{-2}$) in green. Parameters in example are: $k_R = 4.335, k_P = 5, K_M = 15.5, \delta_P = 0.05, \delta_R = 0.17$.

B Supplementary methods

B.1 GRN Analysis and Fitting

To inform our selection of appropriate GRNs to study, we approximated connectivity parameters from a statistical analysis of available human genomic data from the literature and publicly available databases.

Connectivity data was compiled from six databases: JASPAR [10], TRANSFAC [11], the Ensembl Regulatory Build [12], TRRUST [13], the regulatory database from *Neph, et al* [14], and miRNA connections (with experimental evidence) from miRTarBase [15]. In- and out-degrees for miRNA-mRNA connections were calculated, as well as in- and out-degrees for TF-DNA connections (Figure S8). Connection counts for each species type were constructed using binomial approximations to the empirical degree distributions. General genome sizes (TF only) and transcript distributions were determined by analyzing processed data from 104 mRNA-seq experiments of human primary and stem cell lines available through the ENCODE project [16] (Figures S11, S12 and S13). Using these data, we identified suitable parameters to randomly generate "human genome-like" genomes; by examining phenomena on a wide range of such GRNs, we believe that it is reasonable that gross qualitative phenomena are likely applicable to the "true" human GRN.

B.2 Parameter estimation

We based our initial parameter choices on published literature whenever possible; when multiple corroborating sources could not be identified, we used the simplest possible assumptions.

Because our initial assumptions are that miRNAs act purely by sequestration, the mRNA degradation rate should be roughly constant. Estimates for rates of mRNA and TF synthesis and decay rates were based off of supplementary data from [7, 17–19] and calibrated to match total mRNA transcript counts from *Islam, 2011* [20]. It has been observed that mouse ES cells contain roughly 10^4 transcripts and express half of their total genes, suggesting a baseline quantity of one mRNA per (transcribable) gene [20].

In this model, TF association and dissociation rates are assumed to be approximately constant as well as mRNA-miRNA binding rates. Because many experimental methods to quantify RNA/TF levels estimate relative (rather than absolute) abundance, the number of studies measuring absolute molecule counts for large numbers of RNAs and TFs in a single cell are somewhat limited. The selection of these parameters was based off of the rates and equilibrium constants found in [21–23]. Rather than taking association and dissociation rates, the network generator takes equilibrium quantities and event frequencies as parameters. This allows us to use equilibrium levels as a parameter, and modulate the time taken to execute reactions. The conventional association and dissociation constants can be calculated as $k_a = F\frac{A}{1+A}$, $k_d = F\frac{1}{1+A}$, where A is the affinity and F is the frequency. It has been reported that functional activation of a target gene requires on the order of 10^3-10^5 TF molecules in total [17], which is supported by several other experiments (reviewed in [24]), suggesting potential binding equilibrium rates. Using the measurements from [21], we calculated approximate association and dissociation rates of 2×10^{-4} and 2. Assuming that roughly $\frac{1}{3}$ of target mRNAs are bound at equilibrium (e.g. from [8, 22], and that miRNA binding equilibrium is reached in about 1 minute, we selected a frequency which was four times that of TFs.

B.3 Identification of time-to-differentiation

Although we used a relatively abstract definition of differentiation, sample simulation trajectories show that one can clearly see that these two phases exist (Figure reffig:sample-traces).

To measure the time to differentiation, we use the Hilbert transform (HT) of the trajectories. The HT is an invertible linear transformation on real-valued L^2 functions on the real line. For a function $f : \mathbb{R} \to \mathbb{R}$, $\mathcal{H}(f)(x) = g(x)$, where $F = f + ig$ is holomorphic; that is, the HT maps a function to its harmonic conjugate.

The Cauchy-Riemann equations imply that an inflection point in the original function is also a root of the derivative of the harmonic conjugate.

The closely-related discrete HT has both real and imaginary parts - for a real-valued signal, the real part generates an envelope for the original signal, while the imaginary part gives the "actual" HT. Use of this transformation is especially useful for a stochastic (real-valued) signal, as it reconstructs the signal by removing certain high-frequency oscillations. The calculation of time-to-differentiation is given by the following steps:

1. Scale each copy number trajectory by its ℓ^2 norm.
2. Sum the values of the scaled trajectories.
3. Apply the DHT to the summed trajectory.
4. Find the time when the imaginary part of the DHT is minimized.

B.4 Simulation Process

The number of GRNs generated and tested at each network size with zero initial conditions is given in Table S4. Experiments modulating initial TF quantities were performed at levels of 10^2, 10^3, 5×10^3, 10^4, 10^5 and 5×10^5 using networks of 100 TFs and active miRNA levels of 0, 10, 20, 30, and 40. One hundred generated networks were tested in total, with 25 trials run at each TF- and miRNA level, corresponding to a total of 75000 simulations in total. In order to balance computational time with the need to run a large number of simulations with a large number of networks, the main results presented were derived from simulations of randomly generated GRNs with 150 TF-coding and 100 miRNA-coding genes (not all necessarily active at once). With the exception of the miRNA-absent simulations, the set of active miRNAs was randomly selected from the pool of 100 pre-generated miRNAs: the remainder were inactivated. A schematic of the active miRNA selection process can be found in Figure S10.

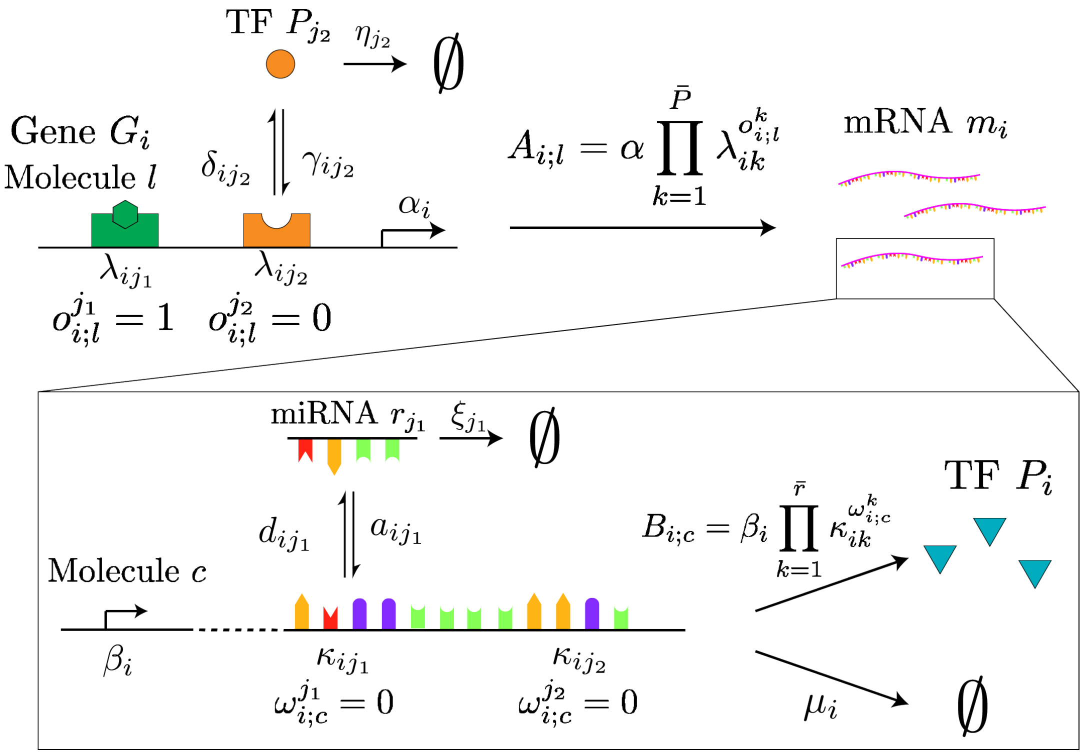

Fig S2. Detailed depiction of stochastic transcription-translation model with propensities using simplified dynamics. Gene i, molecule l ($G_{i;l}$) has a set of binding sites for TFs (denoted here as P_{j_1}, P_{j_2}) with corresponding occupancies $o^j_{i;l}$ and effects λ_{ij}. TFs associate/dissociate with constants γ, δ, and decay with rates η (subscripts suppressed). Transcription propensity per molecule ($A_{i;l}$) is the product of the basal transcription rate and the effects of each bound TF. Transcription behaves identically for both TF-coding and miRNA-coding genes. Below, the transcription product, mRNA i, molecule c ($m_{i;c}$) has a very similar structure to $G_{i;l}$, with occupancies and effects (denoted ω, κ) for each bound miRNA, and miRNA association/dissociation rates denoted by a, d. Translation rate $B_{i;c}$ is constructed in an identical manner to transcription. MiRNAs can decay with rates ξ while mRNAs decay with rates η.

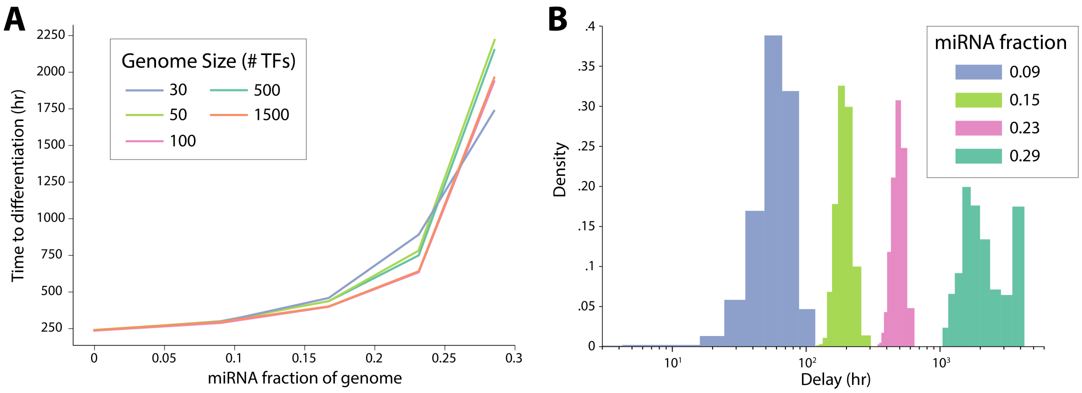

Fig S3. Relative and absolute control of differentiation time by miRNAs. **A**) the effect of miRNAs on time to differentiation is independent of genome size. **B**) histogram of delay times (differences of trials with miRNAs present and trials with miRNAs absent) shows a clear separation of delay magnitudes by miRNA fraction. Note that for miRNA fractions below .29, the range of delay times within each group is roughly equal to its median. For the highest fraction (.29, *red*), the delay time range is roughly three times its median, suggesting a breakdown in the predictability of network dynamics with high miRNA content.

Table S1. Studies supporting the necessity for miRNA-mediated gene regulation in the development of different organ systems and organisms. Note that these are not studies identifying miRNAs in organisms; rather, they are experiments showing that elimination of some or all miRNAs in a particular organ/tissue type results in failure to develop, survive, and/or reproduce.

Organ System	Reference(s)
Hematopoiesis	Kumar et al, Blood 2015 [25]
	Raaijmakers et al, Blood 2009 [26]
Thyroid development	Rodriguez et al, PLoS One 2012 [27]
Cardiac development	Martins et al, Circulation 2008 [28]
	Chen et al, PNAS 2008 [29]
Limb development	Harfe et al, PNAS 2005 [30]
Oogenesis	Nagaraja et al, Mol Endocrinol 2008 [31]
Spermatogenesis	Hayashi et al, PLoS One 2008 [32]
Angiogenesis	Yang et al, J Biol Chem 2005 [33]
Neurogenesis	Kawase-Koga et al, Dev Dyn 2009 [34]
Brain development	Giraldez et al, Science 2005 [35]

Organism	Reference(s)
H. sapiens	Walz et al, Cancer cell 2015 [36]
M. musculus	Park et al, Hum. Mol. Genet. 2010 [37]
C. elegans	Reinhart et al, Nature 2000 [38]
D. melanogaster	Wheeler et al, Evol. Dev. 2009 [39]
D. rerio	Nagaraja et al, Mol Endocrinol 2008 [31]
A. thaliana	Vaucheret et al, Genes Dev. 2004 [40]

Table S2. Default values for network generation parameters for results in section 4.1. Note that for connectivities, there are three copies of the parameters $d_l,\ d_h,\ p$, and $inOut$ corresponding to miRNA/mRNA, TF/TF-coding DNA, and TF/non-coding DNA interaction types. Total connection counts are binomially distributed as Binom (d_l, d_h, p). For TF effect strengths λ, they are determined using a uniform distribution $U(\lambda_{min}, \lambda_{max})$. These parameters only specify general attributes, with each network generated pseudorandomly. See GitHub repository for more information.

Type	Name	Default value	Description
Dimensions	$nMess$	150	# of mRNAs (alt. # TFs)
	$nMicro$	100	Total # miRNA species in generated network
Connectivities	d_l	0, 0, 0	minimum degree
	d_h	50, 50, 50	maximum degree
	p	$\{.1, .2, .3, .4, .5\}$	probability of connection (randomly chosen)
	$inOut$	$in,\ in,\ in$	Whether conn. params specify in- or outdegree
mRNA	α	$1.0/hr$	Basal Transcription rate
	μ	$1.0/hr$	mRNA decay rate
TFs	β	$100/hr$	Translation rate (per transcript)
	η	$10^{-2}/hr$	Decay rate
	$Affinity$	1×10^{-4}	TF-DNA equilibrium constant
	$Frequency$	1	TF-DNA event frequency
	λ_{min}	0.5	Minimal TF effect
	λ_{max}	3.0	Maximal TF effect
miRNAs	α	$1.0/hr$	miRNA transcription rate
	ξ	$1.0/hr$	miRNA decay rate
	$Affinity$	0.33	miRNA-mRNA equilibrium const
	$Frequency$	4	miRNA-mRNA event frequency
	κ	10^{-2}	miRNA effect strength
ICs	C_0	2	TF-coding DNA copy #
	nC_0	8	miRNA-coding DNA copy #
	R_0	0	Initial mRNA transcript copy #
	r_0	0	Initial miRNA transcript copy #
	P_0	0	Initial TF copy #

Table S3. Default values for network generation parameters in section 4.2. Indegrees are drawn from clamped, displaced negative binomial distributions specified by N_f, p, d_l, d_h, where the three values listed correspond to miRNA/mRNA, TF/TF-coding DNA, and TF/non-coding DNA interaction types (see also Figure S8). TF effect strengths are chosen from a lognormal distribution with parameters m, s corresponding to the mean and standard deviation of the underlying normal distribution. Note that the miRNA modulatory variables κ, μ are not listed here because they are varied, see Table S5.
In this formulation, the connectivity distribution is given as:

$$\Pr\left(k; N_f, p, d_l, d_h\right) = \begin{cases} 0, k < d_l \text{ or } k > d_h \\ \binom{k+r-(d+1)}{k-d_l} p^{k-d_l} \left(1-p\right)^{N_f}, d_l \leq k < d_h \\ \sum_{l=d_h}^{\infty} \binom{l+r-(d_l+1)}{l-d_l} p^{l-d_l} \left(1-p\right)^{N_f}, k = d_h \end{cases}$$

Type	Name	Default value	Description
Dimensions	$nMess$	150	# of mRNAs (alt. # TFs)
	$nMicro$	100	Total # miRNA species in generated network
Connectivities	N_f	$.7966, 2.052, 2.4027$	# Number of failures
	p	$.0430,\ .1069,\ .1770$	“success” probability
	d_l	$0, 2, 1$	min degree
	d_h	$nMicro, nMess, nMess$	max degree
	$inOut$	$in,\ in,\ in$	Whether conn. params specify in- or outdegree
mRNA	α	$1.0/hr$	Basal Transcription rate
	μ	$1.0\left(K_D+1\right)/hr$	mRNA decay rate
TFs	β	$50\left(K_L+1\right)/hr$	Translation rate (per transcript)
	η	$10^{-2}/hr$	Decay rate
	$Affinity$	1×10^{-4}	TF-DNA equilibrium constant
	$Frequency$	1	TF-DNA event frequency
miRNAs	α	$1.0\left(K_C+1\right)/hr$	miRNA transcription rate
	ξ	$1.0/hr$	miRNA decay rate
	$Affinity$	0.33	miRNA-mRNA equilibrium const (where applicable)
	$Frequency$	4	miRNA-mRNA event frequency (where applicable)
ICs	C_0	2	TF-coding DNA copy #
	nC_0	8	miRNA-coding DNA copy #
	R_0	0	Initial mRNA transcript copy #
	r_0	0	Initial miRNA transcript copy #
	P_0	0	Initial TF copy #

Table S4. Number of networks generated and simulated at each size for the results in section 4. Baseline miRNAs represent the total pool of *potential* active miRNAs in system. For each simulation run, a random subset of this pool was activated and tested. Subset sizes were 0, 15, 30, 45 and 60. Networks used to generate main results in Section 4 are indicated with an asterisk.

# TFs	# miRNAs (baseline)	# of networks tested
30	20	100
50	35	100
100	66	100
150*	100	1000
500*	350	1000
1500	1000	100

Table S5. Parameter values used for the results in section 4.2. For each combination of values (27648 total), ten networks were generated and tested. Networks with 150 TFs and 100 miRNAs (baseline) were used. For each network, four simulations each were run with 0, 10, 20, 30 and 40 randomly-selected active miRNAs, adding up to a total of approximately 5.53M simulations.

Parameter	Values tested
K_C	0.01, 0.1, 1.0, 6.0, 10.0, 100.0
K_L	0.01, 0.1, 1.0, 6.0, 10.0, 100.0
K_D	0.01, 0.1, 1.0, 6.0, 10.0, 100.0
κ	0, 0.01, 0.1, 0.5
μ	1.5, 2.0, 5.0, 10.0
m	$-.875$, $-.25$, .625, 1.5
s	0.25, 0.75

B.5 Stochastic simulation algorithm

Because genes (DNA) and mRNAs have bound state information on a per-molecule basis (e.g. $\mathbf{o}$ and ω from above), they are treated as individual agents. In the model, miRNAs and TFs do not carry states, so only quantities of each species are tracked. This design is implemented as a hybrid agent-based/population-based model using a modified Gibson-Bruck algorithm. It can simulate large numbers of events ($> 10^{10}$) using "genome-scale" GRNs (up to 10^5 distinct species of protein-coding genes and miRNAs) on a Macbook Pro 2013 in under 1 hour. The system design allows for a very high level of specification and customization, and allows the import and use of "named" genes and miRNAs with individual kinetic parameters.

B.5.1 Simulation code & availability

All code and more information can be found at https://github.com/adpposner/GRNsim. Source code for the simulation is written exclusively in C, while the analysis tools are written in a combination of C and Fortran; all code adheres to the C99 and Fortran 95 standards. However, a variety of platform-specific optimizations and library dependencies have been used in order to maximize performance, and the compilation, execution, and analysis of the base code used in our results requires some specialized knowledge and likely end-user platform-specific modifications.

Nonetheless, we are committed to transparency, availability, and reproducibility of our methods. On the repository, we also host a slightly less-performant but equivalent version of the software that should run on any 64-bit Linux platform using free, open-source tools. This version is accompanied by a Dockerfile which can be used to build a complete, working Docker image on any platform. More information is available in the GitHub repository.

B.5.2 Data model and implementation

As previously mentioned, the stochastic simulation algorithm treats molecular species and reactions using a hybrid individual-population method which is run using a modified Gibson-Bruck algorithm (GBA). The GBA is a stochastic simulation which depends on two data structures - a reaction priority queue (PQ) and a graph of reaction dependencies. The priority queue pairs each reaction with its theoretical time to execution. This execution time is selected pseudorandomly from an exponential distribution with rate parameter equal to the reaction propensity. This value is used as the key to order the PQ. After initialization, the general series of steps are:

1. Execute reaction r at top of PQ
2. For each reaction which is dependent on r, scale its time to execution based on its new rate and previous execution time.
3. Add the r's execution time to the current "clock time"
4. Update the PQ with the new reaction/reaction time pairs.

The precise circumstances under which this algorithm outperforms other stochastic simulation algorithms has been studied extensively, but it is worth noting that for networks with large numbers of reactions, the GBA does not require the serial addition and subtraction of many floating-point numbers, and is useful when the search depth of the reaction array is high.

However, our application has two important distinctions. The first is that there are really only four types of reactions: synthesis (transcription or translation), decay, association and dissociation. This allows us to use a very simple dependency graph, since we know what types of reactions affect which others. In fact, in almost all cases, we not only know the relationships between different reactions, but also know the direction in which each reaction changes the rate of its dependencies. In the GBA, this means that we do not have to compare each element in the PQ with its predecessor and child elements, but rather can pick a single direction to do comparisons. This set of dependencies and and rate changes is depicted in Table S6.

The stochastic simulation relies on a number of different optimizations, but probably the most important optimization is to recognize two essential facets of our reaction system: there are only four reaction types and two species types. The reaction types can be broken into two complementary pairs.

Synthesis & Decay Three types of molecules can be synthesized: mRNAs, miRNAs, and TFs. These are produced by coding DNA, noncoding DNA, and mRNA, respectively. The three types of molecules that can be synthesized - mRNA, miRNA, and TF, are also capable of decay. Only free (unbound) miRNA and TF are capable of spontaneous decay. When an mRNA molecule decays, miRNA molecules which were bound to it either also decay or are released into the free miRNA pool. TF bound to DNA must dissociate prior to its decay.

Association & Dissociation Association and dissociation reactions are limited to interactions between either TF and DNA, or miRNA and mRNA. When TF binds to a molecule of DNA, it modulates the synthesis (transcriptional) rate of its target. This modulation is bidirectional; a TF can either increase or decrease synthesis rates. When miRNA binds to a molecule of mRNA, it modulates the synthesis (translational) rate of its target. This modulation is unidirectional; miRNAs can only decrease synthesis rates. Depending on the particular rule set used, a bound miRNA may or may not increase the decay rate of its mRNA target.

As noted above, the only reactions whose propensities depend on binding configurations are the synthesis reactions - transcription and translation - and mRNA decay. Thus, it is essential to keep track of the configurations of these species, as well as molecule counts (for mRNA species). This division leads to a natural partition of the chemical species in the network into two categories: the *Producers*, which are capable of carrying out synthesis reactions, and the *Modulators*, which bind to Producers to alter their reaction rates.

Modulators The modulator species are those which can alter synthesis/decay rates through association and dissociation reactions. They are effectively indistinguishable, meaning that only their total counts need to be stored at each time point.

Producers The producer molecular species are those which are capable of executing synthesis reactions. These are: TF-coding and miRNA-coding DNA and mRNA. The copy numbers of the DNA species are fixed, while those of mRNA are variable. However, the binding of TF or miRNA to these elements define their synthesis and decay (mRNA only) rates. Therefore, the state of each molecule must be recorded. The computational organization of the molecular states is depicted in Figure S5. Synthesis reactions are fairly straightforward, while a decay reaction is illustrated in Figure S6. Association/dissociation reactions are illustrated in Figure S7.

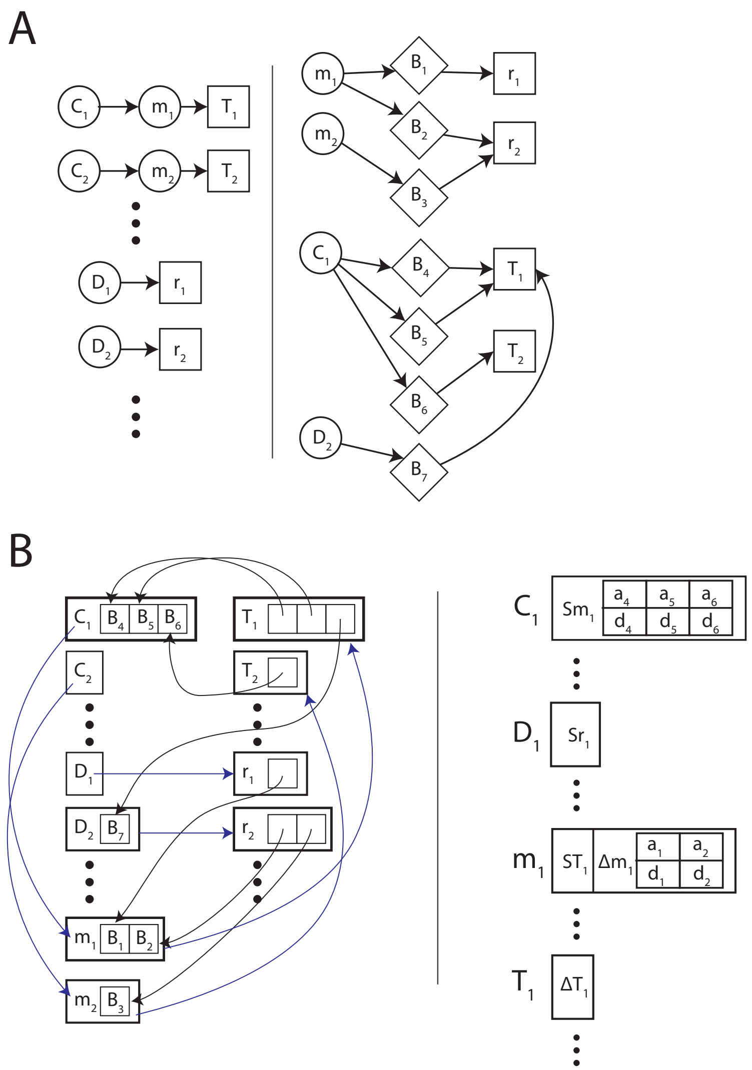

Fig S4. **A**) Input to the simulator consists of a synthesis graph (*left*) and an interaction graph (*right*). The interaction graph contains intermediate bound elements, indexed as B_i, each of which corresponds to a single (Producer,Modulator) pair. Note that multiple binding sites are built by constructing duplicate pairs - both B_4 and B_5 correspond to the binding of coding gene C_1 and TF T_1. **B**) This input is used to build a compact in-memory representation of an entity (*left*) and reaction (*right*) diagram. Bound elements exist as virtual entities which are owned by their respective producer, while modulators hold locations of those bound entities; producers hold locations of their products as well (blue arrows). This structure minimizes the overhead of calculating synthesis and catalyzed decay rates. The reaction diagram is ordered linearly. For each entity, reactions are ordered: synthesis, decay, association reactions and dissociation reactions (prefixed with S,Δ, a, and d, respectively). The hierarchical ordering of reaction blocks (DNA → mRNA → TF → miRNA reactions. The letters C and D are used to distinguish TF-coding and miRNA-coding DNA, respectively.

	mRNA TxC	miRNA TxC	TxL	mRNA decay	miRNA decay	TF decay	RNA assoc	RNA dissoc	DNA assoc	DNA dissoc
mRNA TxC	=		↑	↑			↑			
miRNA TxC		=			↑		↑			
TxL			=			↑			↑	
mRNA decay			↓	↓	↑**		↑**	↓		
Free miRNA decay					↓		↓			
Free TF decay						↓			↓	
RNA assoc			↓	↑*	↓		↓	↑		
RNA dissoc			↑	↓*	↑		↑	↓		
DNA assoc	↕	↕				↓			↓	↑
DNA dissoc	↕	↕				↑			↑	↓

* effect only if catalytic decay active

** effect only if miRNAs are recycled

Table S6. Because the number of types of reactions is limited, the dependency graph structure is greatly simplified and the reaction list can be efficiently indexed to minimize memory fetch instructions. Furthermore, when a reaction is triggered (*rows*), the direction of change for nearly all dependent reactions rate (*columns*) can be anticipated, minimizing comparisons in the PQ. It is important to note that when miRNAs are recycled, each bound miRNA which is returned to the pool is equivalent to being transcribed anew, and a series of cascading updates must be performed on each miRNA which is freed, greatly expanding the number of dependencies. Arrows indicate direction of change, dual-direction arrow indicates unknown direction, = indicates no change in reaction rate.

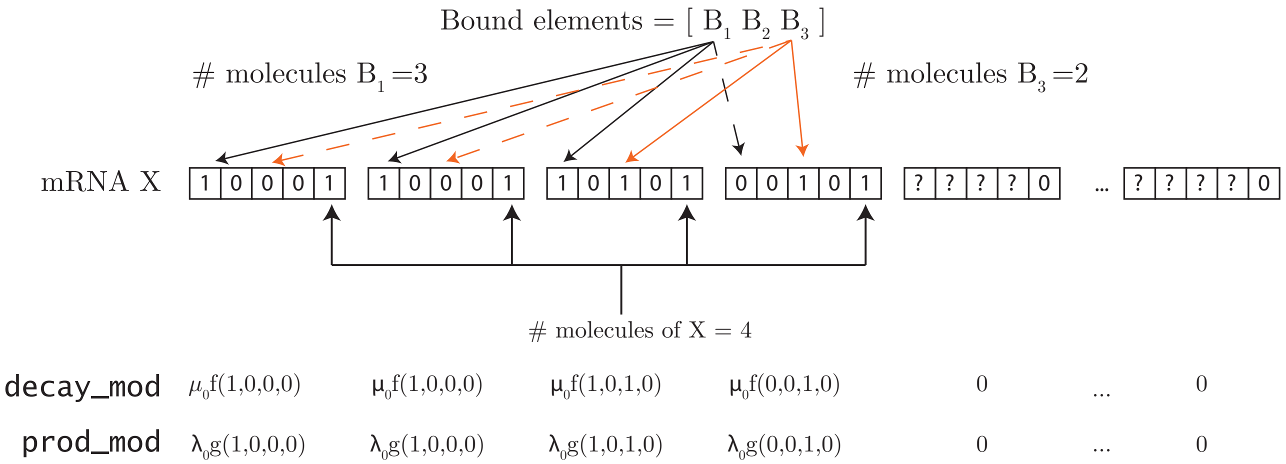

Fig S5. In-memory representation of state for hypothetical mRNA "X". The state of each molecule is represented by a string of bits, where the least significant bit (LSB) is set for each extant molecule. Bound elements (B_i) are ordered and assigned indices for an entity. If a molecule has its nth most significant bit (MSB) set, that molecule bound to A_n's corresponding Modulator (solid lines indicate bound molecules, dashed lines indicate available binding slots). Each bitset (molecule) stores its own "production" and "decay" (mRNA only) modifiers, which are functions of the molecule's state. The total synthesis and decay propensity for mRNA X is the sum of these modifiers.

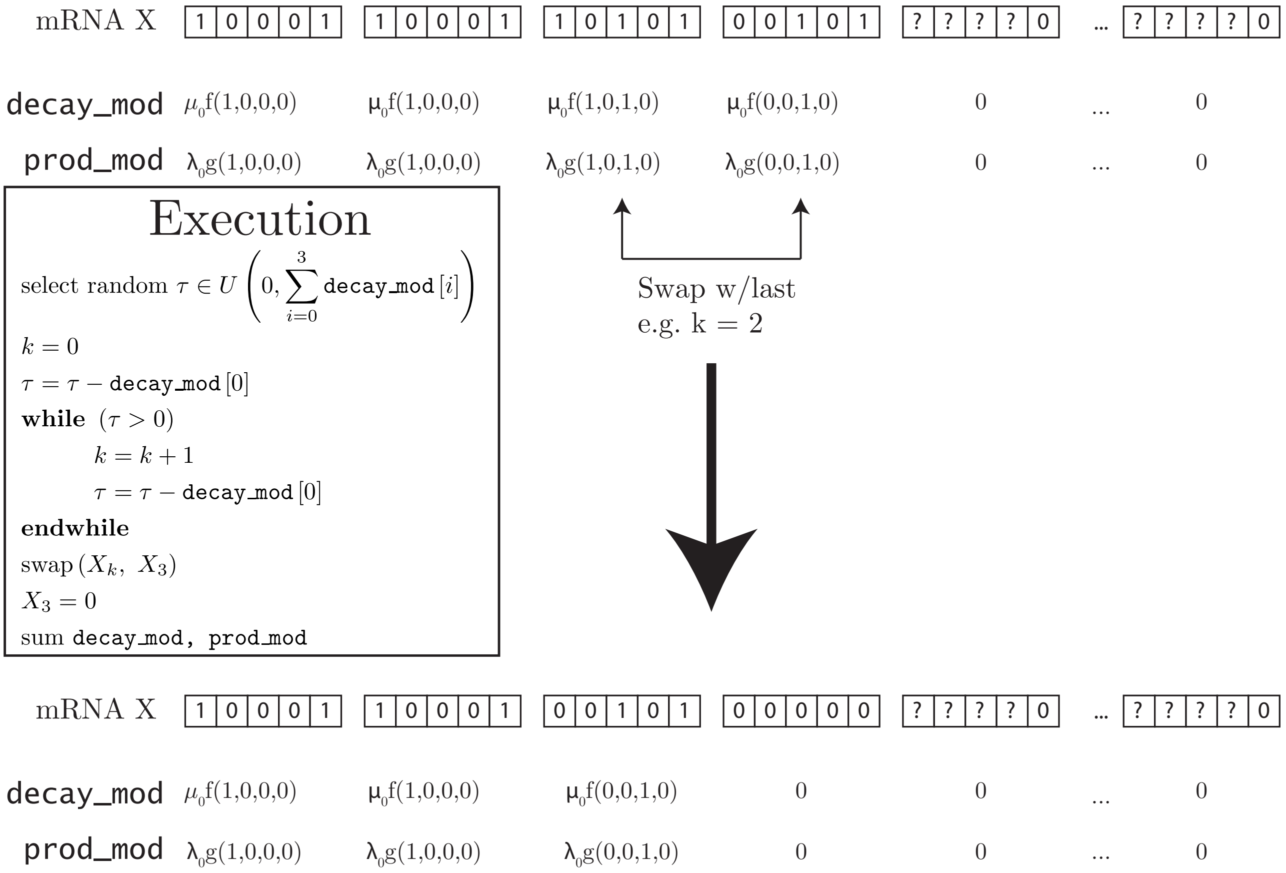

Fig S6. Illustration of the "execution" of a decay reaction for mRNA X with an initial quantity of 4 molecules. A number $\tau \in (0, p_D)$ is drawn uniformly, where p_D is the total decay propensity. The routine walks down the array of molecules starting at molecule $k = 0$, subtracting individual decay propensities from τ until $\tau < 0$ ($k = 2$ in example). The state of the $4^t h$ molecule in the array is swapped with the $(k+1)^{th}$ molecule, and the LSB of the 5th molecule is cleared. Depending on model chosen, either forced dissociation reactions or forced decay reactions are executed for the remaining set bits on the 4th bitset. Finally, translation and decay propensities are recalculated.

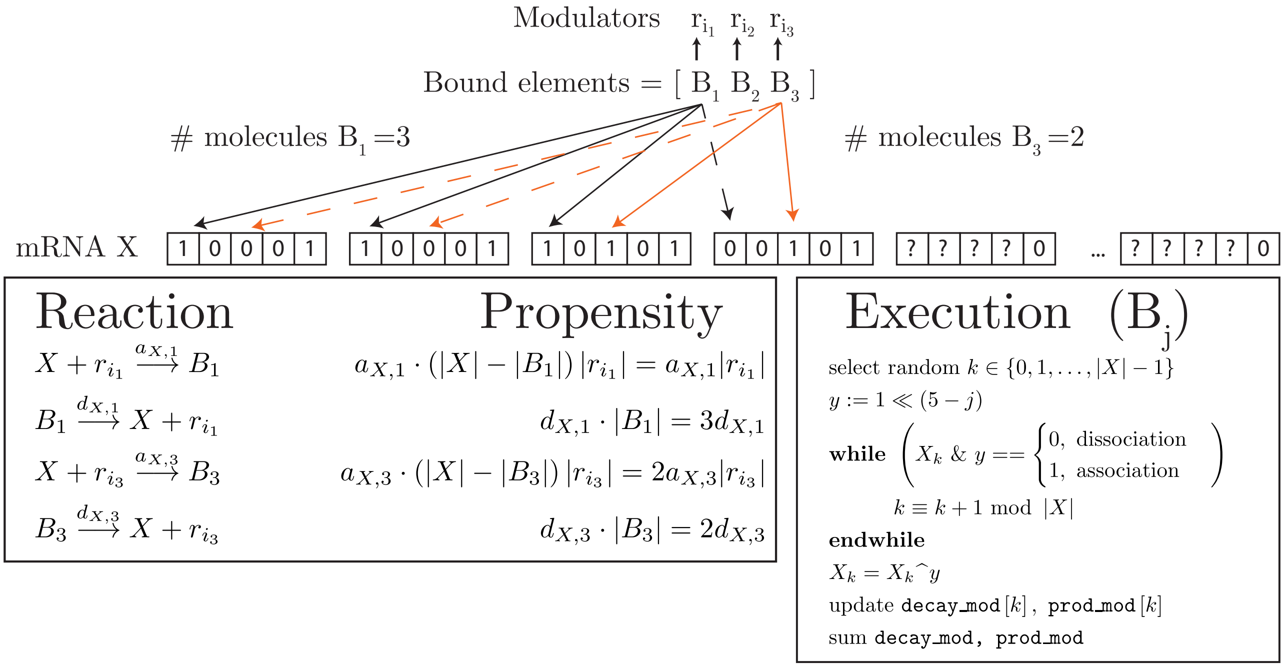

Fig S7. Execution of association and dissociation reactions for mRNA X. Dissociation propensities depend on the number of bound elements for each index (solid lines), while association propensities depend on the number of available slots (dashed lines). To execute a reaction involving bound element A_n, a random number k is drawn in $\{0, 1, \ldots, |X - 1|\}$. Beginning with the molecule at index k, the routine begins at the $k+1^{th}$ molecule and walks across each bitset (modulo $|X|$) until it finds a molecule whose n^{th} MSB is clear (association) or set (dissociation). The nth MSB is toggled, and the molecule's production/decay modifier is recalculated, and production/decay modifiers are summed up to obtain the new production/decay propensity.

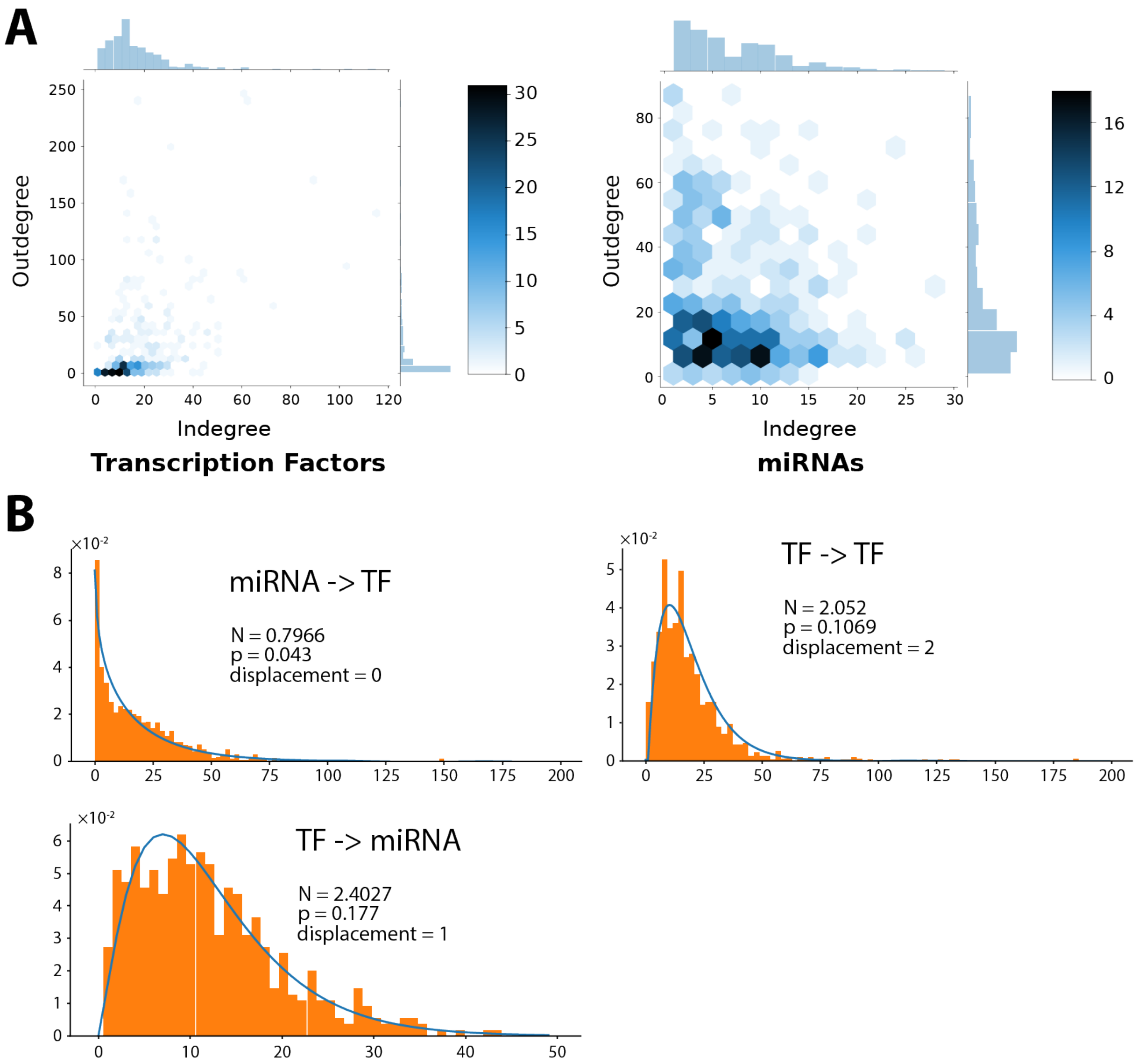

Fig S8. Plot of distributions of in- and outdegrees for TFs and miRNAs compiled from databases noted in *Methods, GRN Analysis & Fitting*. **A**) hexplot of in and outdegrees. Note that for TFs, in and out-elements can be either TFs or miRNAs, while for miRNAs, both in- and out-elements are TFs only. **B**) MLE of in and outdegrees using a (clamped and displaced) negative binomial distribution with parameters, used in section 4.2.

Fig S9. Trace examples (upper, 5 randomly chosen TFs) shows that # of miRNAs can produce a visible delay in "time-to-differentiation" - however, one needs an objective, computational approach. Note that at some point - approximately 200 hr in the 0 miRNA case and 2000 hr in the 30 miRNA case, the traces, when shifted down, resemble odd curves. After scaling each trace by its 2-norm, summing the scaled values, and applying the Hilbert transform (lower), "oddness" corresponds to a minimum of the imaginary part (lower, red) of the discrete HT, which we used to identify differentiation time.

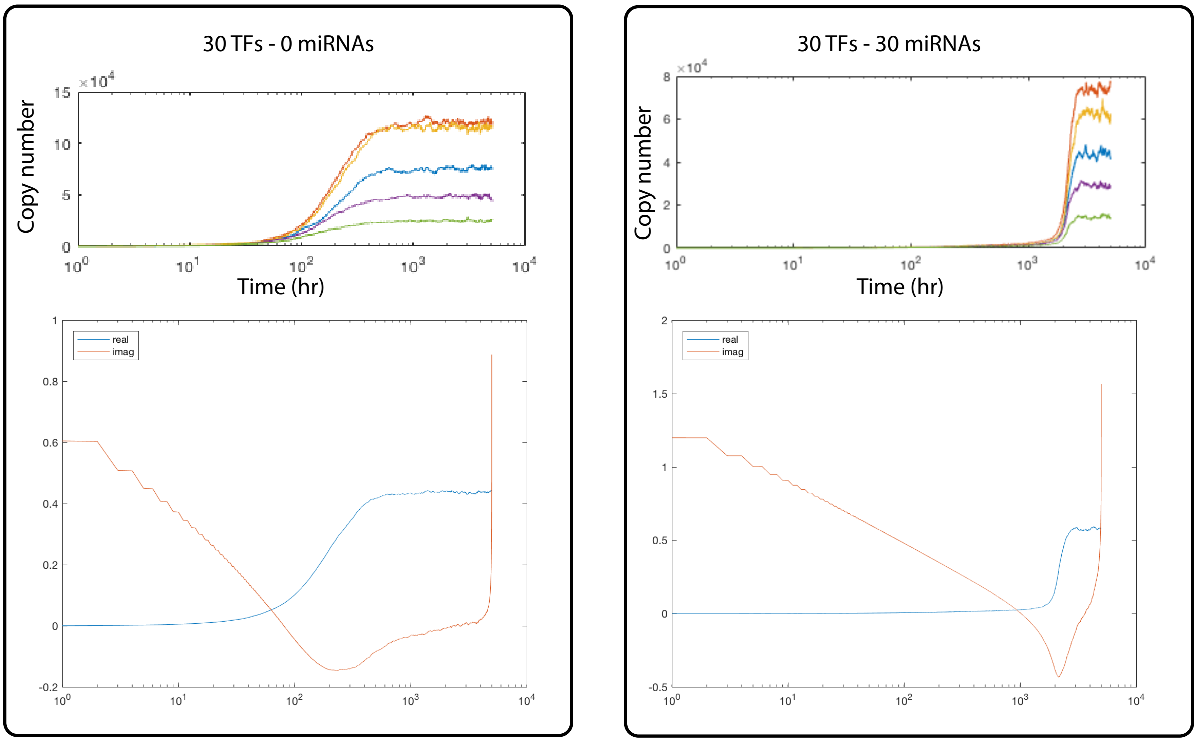

Fig S10. Example of simulation process. **A**) all TFs (blue circles) and 0 miRNAs are active (inactive elements shown greyed out). For each simulation for a given quantity of active miRNAs, the appropriate number of miRNAs were chosen randomly from the network and activated - in this example, we have shown levels of 25% (**B**), 50% (**C**), and 75% (**D**) active miRNAs from original network (inset).

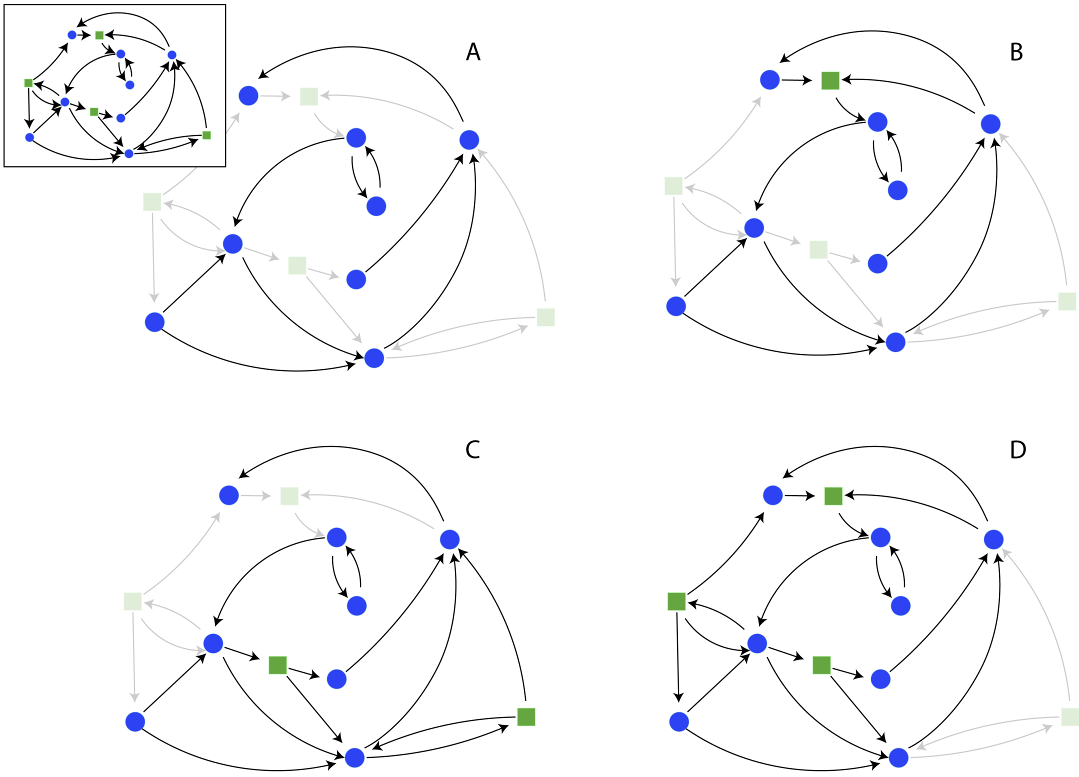

Fig S11. Number of TF genes expressed at ≥ 1 transcript per million (TPM) across different cell types shows that stem cells express many more TFs than other, more differentiated cell types. Below, although the number of different genes transcribed is much higher, TPM counts by gene are much lower.

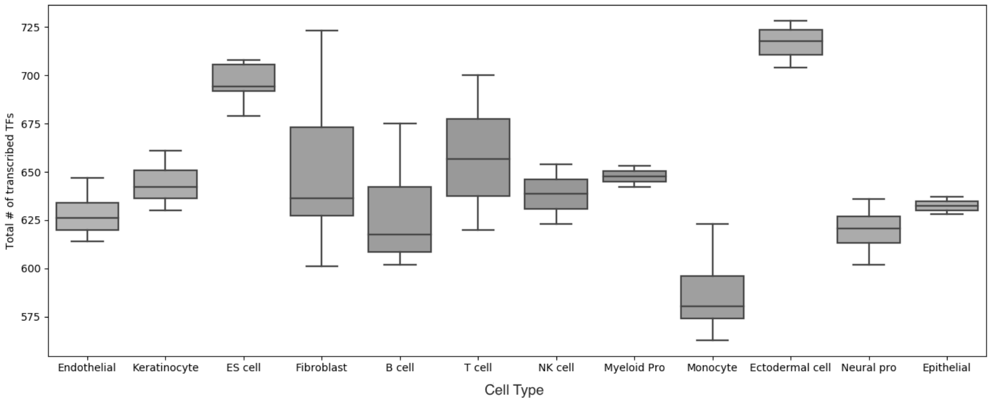

Fig S12. Distribution of transcripts per gene (measured as transcripts per million, TPM) by cell type from ENCODE data. Despite the high number of TFs expressed by ES cells (lower right), one can see that more than 95% of TFs are expressed at fewer than 5 TPM.

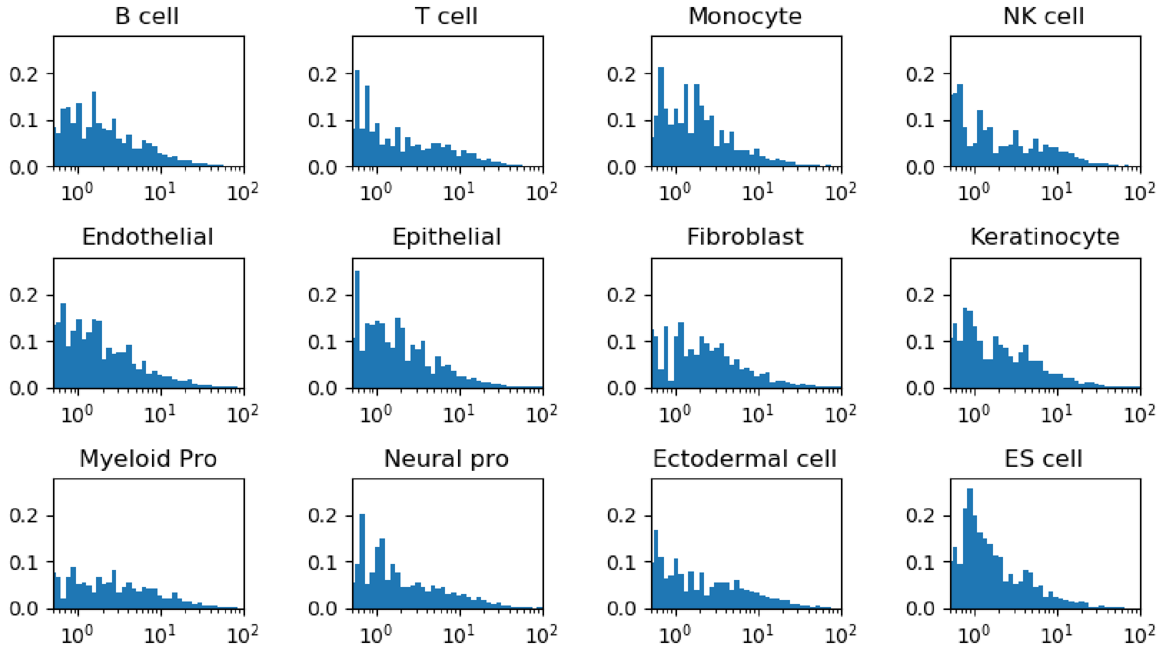

Fig S13. Expression of argonaute genes remains relatively consistent across cell types. The argonaute proteins, particularly AGO2, are critical for formation of the RNA-induced silencing complex (RISC)[41]. Their relative constancy across cell types suggests that the number of formed RISC complexes possibly saturates at a number well below the number of ribosomes in a typical mammalian cell ($10^6 - 10^7$) [42].

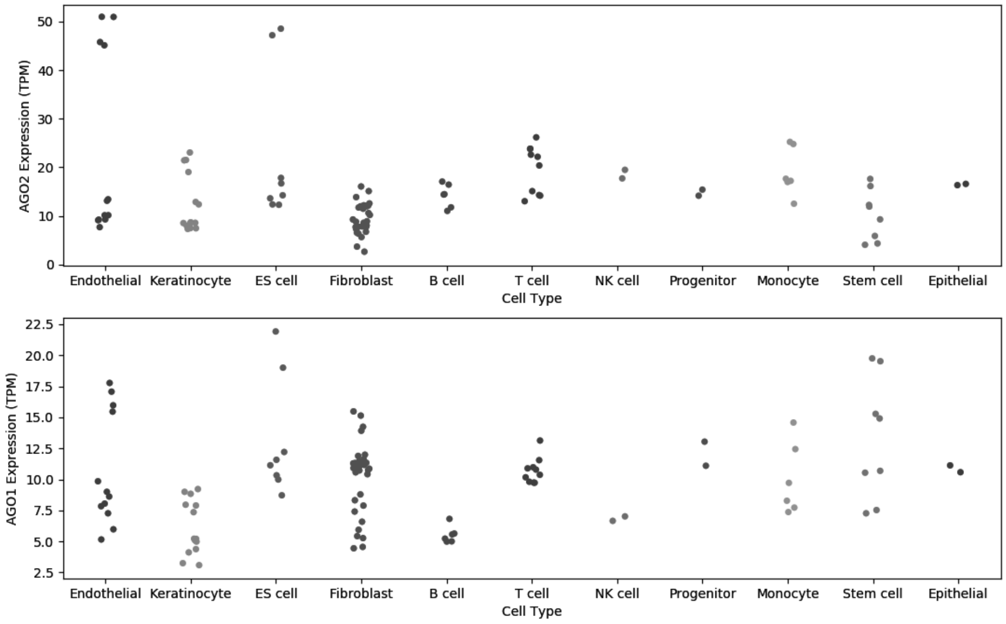

References

[1] Marco Del Giudice et al. "On the role of extrinsic noise in microRNA-mediated bimodal gene expression". *PLoS Comput Biol* 14.4 (Apr. 17, 2018). DOI: 10.1371/journal.pcbi.1006063.

[2] Velia Siciliano et al. "miRNAs confer phenotypic robustness to gene networks by suppressing biological noise". *Nature Communications* 4 (Sept. 30, 2013), p. 2364. DOI: 10.1038/ncomms3364.

[3] Karin Lykke-Andersen et al. "Maternal Argonaute 2 Is Essential for Early Mouse Development at the Maternal-Zygotic Transition". *Mol. Biol. Cell* 19.10 (Oct. 1, 2008), pp. 4383–4392. DOI: 10.1091/mbc.E08-02-0219.

[4] Fuchou Tang et al. "Maternal microRNAs are essential for mouse zygotic development". *Genes Dev* 21.6 (Mar. 15, 2007), pp. 644–648. DOI: 10.1101/gad.418707.

[5] Rémy Denzler et al. "Assessing the ceRNA hypothesis with quantitative measurements of miRNA and target abundance". *Mol Cell* 54.5 (June 5, 2014), pp. 766–776. DOI: 10.1016/j.molcel.2014.03.045.

[6] Shankar Mukherji et al. "MicroRNAs can generate thresholds in target gene expression". *Nat Genet* 43.9 (Aug. 21, 2011), pp. 854–859. DOI: 10.1038/ng.905.

[7] Björn Schwanhäusser et al. "Global quantification of mammalian gene expression control". *Nature* 473.7347 (May 19, 2011), pp. 337–342. DOI: 10.1038/nature10098.

[8] Jörn M. Schmiedel et al. "MicroRNA control of protein expression noise". *Science* 348.6230 (Apr. 3, 2015), pp. 128–132. DOI: 10.1126/science.aaa1738.

[9] Matteo Osella et al. "The role of incoherent microRNA-mediated feedforward loops in noise buffering". *PLoS Comput. Biol.* 7.3 (Mar. 2011), e1001101. DOI: 10.1371/journal.pcbi.1001101.

[10] Anthony Mathelier et al. "JASPAR 2014: an extensively expanded and updated open-access database of transcription factor binding profiles". *Nucleic Acids Res.* 42 (Database issue Jan. 2014), pp. D142–147. DOI: 10.1093/nar/gkt997.

[11] V. Matys et al. "TRANSFAC and its module TRANSCompel: transcriptional gene regulation in eukaryotes". *Nucleic Acids Res.* 34 (Database issue Jan. 1, 2006), pp. D108–110. DOI: 10.1093/nar/gkj143.

[12] Daniel R. Zerbino et al. "The Ensembl Regulatory Build". *Genome Biology* 16 (Mar. 24, 2015), p. 56. DOI: 10.1186/s13059-015-0621-5.

[13] Heonjong Han et al. "TRRUST: a reference database of human transcriptional regulatory interactions". *Sci Rep* 5 (June 12, 2015), p. 11432. DOI: 10.1038/srep11432.

[14] Shane Neph et al. "Circuitry and dynamics of human transcription factor regulatory networks". *Cell* 150.6 (Sept. 14, 2012), pp. 1274–1286. DOI: 10.1016/j.cell.2012.04.040.

[15] Chih-Hung Chou et al. "miRTarBase 2016: updates to the experimentally validated miRNA-target interactions database". *Nucleic Acids Res* 44 (D1 Jan. 4, 2016), pp. D239–D247. DOI: 10.1093/nar/gkv1258.

[16] The ENCODE Project Consortium. "A User's Guide to the Encyclopedia of DNA Elements (ENCODE)". *PLOS Biology* 9.4 (Apr. 19, 2011), e1001046. DOI: 10.1371/journal.pbio.1001046.

[17] Jovan Simicevic et al. "Absolute quantification of transcription factors during cellular differentiation using multiplexed targeted proteomics". *Nature Methods* 10.6 (June 2013), pp. 570–576. DOI: 10.1038/nmeth.2441.

[18] Caroline C. Friedel et al. "Conserved principles of mammalian transcriptional regulation revealed by RNA half-life". *Nucleic Acids Res* 37.17 (Sept. 2009), e115. DOI: 10.1093/nar/gkp542.

[19] Jean Hausser et al. "Timescales and bottlenecks in miRNA-dependent gene regulation". *Mol Syst Biol* 9 (Dec. 3, 2013), p. 711. DOI: 10.1038/msb.2013.68.

[20] Saiful Islam et al. "Characterization of the single-cell transcriptional landscape by highly multiplex RNA-seq". *Genome Res* 21.7 (July 2011), pp. 1160–1167. DOI: 10.1101/gr.110882.110.

[21] Johan Elf, Gene-Wei Li, and X. Sunney Xie. "Probing Transcription Factor Dynamics at the Single-Molecule Level in a Living Cell". *Science* 316.5828 (May 25, 2007), pp. 1191–1194. DOI: 10.1126/science.1141967.

[22] Liang Meng Wee et al. "Argonaute Divides Its RNA Guide into Domains with Distinct Functions and RNA-Binding Properties". *Cell* 151.5 (Nov. 21, 2012), pp. 1055–1067. DOI: 10.1016/j.cell.2012.10.036.

[23] Keng Boon Wee et al. "Transcription Factor Oscillations Induce Differential Gene Expressions". *Biophys J* 102.11 (June 6, 2012), pp. 2413–2423. DOI: 10.1016/j.bpj.2012.04.023.

[24] Mark D. Biggin. "Animal Transcription Networks as Highly Connected, Quantitative Continua". *Developmental Cell* 21.4 (Oct. 18, 2011), pp. 611–626. DOI: 10.1016/j.devcel.2011.09.008.

[25] Bijender Kumar et al. "Dicer Ablations in Bone Marrow Niche Impair Hematopoietic Progenitor/Stem Cells and Induce Myelodysplasia in Young Mice but Are Dispensable for Adult Hematopoiesis". *Blood* 126.23 (Dec. 3, 2015), pp. 1196–1196.

[26] Marc HGP Raaijmakers et al. "Niche Induced Myelodysplasia and Secondary Hematopoietic Neoplasia Caused by Deletion of Dicer1 in Osteoprogenitor Cells." *Blood* 114.22 (Nov. 20, 2009), pp. 247–247.

[27] Wendy Rodriguez et al. "Deletion of the RNaseIII Enzyme Dicer in Thyroid Follicular Cells Causes Hypothyroidism with Signs of Neoplastic Alterations". *PLOS ONE* 7.1 (Jan. 5, 2012), e29929. DOI: 10.1371/journal.pone.0029929.

[28] Paula A. da Costa Martins et al. "Conditional dicer gene deletion in the postnatal myocardium provokes spontaneous cardiac remodeling". *Circulation* 118.15 (Oct. 7, 2008), pp. 1567–1576. DOI: 10.1161/CIRCULATIONAHA.108.769984.

[29] Jian-Fu Chen et al. "Targeted deletion of Dicer in the heart leads to dilated cardiomyopathy and heart failure". *PNAS* 105.6 (Feb. 12, 2008), pp. 2111–2116. DOI: 10.1073/pnas.0710228105.

[30] Brian D. Harfe et al. "The RNaseIII enzyme Dicer is required for morphogenesis but not patterning of the vertebrate limb". *Proc. Natl. Acad. Sci. U.S.A.* 102.31 (Aug. 2, 2005), pp. 10898–10903. DOI: 10.1073/pnas.0504834102.

[31] Ankur K. Nagaraja et al. "Deletion of Dicer in Somatic Cells of the Female Reproductive Tract Causes Sterility". *Mol Endocrinol* 22.10 (Oct. 1, 2008), pp. 2336–2352. DOI: 10.1210/me.2008-0142.

[32] Katsuhiko Hayashi et al. "MicroRNA Biogenesis Is Required for Mouse Primordial Germ Cell Development and Spermatogenesis". *PLOS ONE* 3.3 (Mar. 5, 2008), e1738. DOI: 10.1371/journal.pone.0001738.

[33] Wei J. Yang et al. "Dicer Is Required for Embryonic Angiogenesis during Mouse Development". *J. Biol. Chem.* 280.10 (Mar. 11, 2005), pp. 9330–9335. DOI: 10.1074/jbc.M413394200.

[34] Yoko Kawase-Koga, Gaizka Otaegi, and Tao Sun. "Different timings of Dicer deletion affect neurogenesis and gliogenesis in the developing mouse central nervous system". *Dev Dyn* 238.11 (Nov. 2009), pp. 2800–2812. DOI: 10.1002/dvdy.22109.

[35] Antonio J. Giraldez et al. "MicroRNAs Regulate Brain Morphogenesis in Zebrafish". *Science* 308.5723 (May 6, 2005), pp. 833–838. DOI: 10.1126/science.1109020.

[36] Amy L. Walz et al. "Recurrent DGCR8, DROSHA, and SIX Homeodomain Mutations in Favorable Histology Wilms Tumors". *Cancer Cell* 27.2 (Feb. 9, 2015), pp. 286–297. DOI: 10.1016/j.ccell.2015.01.003.

[37] Chong Y. Park, Yun S. Choi, and Michael T. McManus. "Analysis of microRNA knockouts in mice". *Hum. Mol. Genet.* 19 (R2 Oct. 15, 2010), R169–R175. DOI: 10.1093/hmg/ddq367.

[38] Brenda J. Reinhart et al. "The 21-nucleotide *let-7* RNA regulates developmental timing in ¡i¿ Caenorhabditis elegans¡/i¿". *Nature* 403.6772 (Feb. 2000), p. 901. DOI: 10.1038/35002607.

[39] Benjamin M. Wheeler et al. "The deep evolution of metazoan microRNAs". *Evolution & Development* 11.1 (Jan. 1, 2009), pp. 50–68. DOI: 10.1111/j.1525-142X.2008.00302.x.

[40] Hervé Vaucheret et al. "The action of ARGONAUTE1 in the miRNA pathway and its regulation by the miRNA pathway are crucial for plant development". *Genes Dev.* 18.10 (May 15, 2004), pp. 1187–1197. DOI: 10.1101/gad.1201404.

[41] Minju Ha and V. Narry Kim. "Regulation of microRNA biogenesis". *Nat Rev Mol Cell Biol* 15.8 (Aug. 2014), pp. 509–524. DOI: 10.1038/nrm3838.

[42] Benjamin Lewin. *Genes VII*. 7th ed. edition. Oxford ; New York: Oxford University Press, Dec. 9, 1999. 990 pp.
